# Prevalence, risk factors, and antimicrobial resistance of endemic healthcare-associated infections in Africa: a systematic review and meta-analysis

**DOI:** 10.1186/s12879-024-09038-0

**Published:** 2024-02-02

**Authors:** Gabriel Kambale Bunduki, Effita Masoamphambe, Tilly Fox, Janelisa Musaya, Patrick Musicha, Nicholas Feasey

**Affiliations:** 1 Malawi-Liverpool-Wellcome Programme, Kamuzu University of Health Sciences, Blantyre, Malawi; 2 Department of Clinical Sciences, Liverpool School of Tropical Medicine, Liverpool, UK; 3 Centre d’Excellence en Maladies Infectieuses et Soins Critiques du Graben (CEMISoCG), Faculty of Medicine, Université Catholique du Graben, Butembo, Democratic Republic of the Congo; 4 School of Medicine, University of St Andrews, St Andrews, UK

**Keywords:** Healthcare-associated infection, Pneumonia, Bloodstream, Surgical site infection, Urinary tract infection, Africa

## Abstract

**Background:**

Healthcare-associated infections (HCAI) place a significant burden on healthcare systems globally. This systematic review and meta-analysis aimed to investigate the prevalence, risk factors, and aetiologic agents of endemic HCAI in Africa.

**Methods:**

MEDLINE/PubMed, CINAHL, and Global Health databases (EBSCOhost interface) were searched for studies published in English and French describing HCAI in Africa from 2010 to 2022. We extracted data on prevalence of HCAI, risk factors, aetiologic agents, and associated antimicrobial resistance patterns. We used random-effects models to estimate parameter values with 95% confidence intervals for risk factors associated with HCAI. This study was registered in PROSPERO (CRD42022374559) and followed PRISMA 2020 guidelines.

**Results:**

Of 2541 records screened, 92 were included, comprising data from 81,968 patients. Prevalence of HCAI varied between 1.6 and 90.2% with a median of 15% across studies. Heterogeneity (*I*^*2*^) varied from 93 to 99%. Contaminated wound (OR: 1.75, 95% CI: 1.31–2.19), long hospital stay (OR: 1.39, 95% CI: 0.92–1.80), urinary catheter (OR: 1.57, 95% CI: 0.35–2.78), intubation and ventilation (OR: 1.53, 95% CI: 0.85–2.22), vascular catheters (OR: 1.49, 95% CI: 0.52–2.45) were among risk factors associated with HCAI. Bacteria reported from included studies comprised 6463 isolates, with *E. coli* (18.3%, *n* = 1182), *S. aureus* (17.3%, *n* = 1118), *Klebsiella* spp. (17.2%, *n* = 1115), *Pseudomonas* spp. (10.3%, *n* = 671), and *Acinetobacter* spp. (6.8%, *n* = 438) being most common. Resistance to multiple antibiotics was common; 70.3% (IQR: 50–100) of Enterobacterales were 3rd -generation cephalosporin resistant, 70.5% (IQR: 58.8–80.3) of *S. aureus* were methicillin resistant and 55% (IQR: 27.3–81.3) *Pseudomonas* spp. were resistant to all agents tested.

**Conclusions:**

HCAI is a greater problem in Africa than other regions, however, there remains a paucity of data to guide local action. There is a clear need to develop and validate sustainable HCAI definitions in Africa to support the implementation of routine HCAI surveillance and inform implementation of context appropriate infection prevention and control strategies.

**Supplementary Information:**

The online version contains supplementary material available at 10.1186/s12879-024-09038-0.

## Panel: research in context

### Evidence before this study

Data describing the burden of endemic healthcare-associated infections (HCAI) and associated antimicrobial resistance (AMR) profile in Africa remain few. In most African countries, HCAI surveillance is not prioritised. The 2009 systematic review of endemic HCAI in Africa included 19 studies, which showed that prevalence of HCAI varied considerably, ranging from 2.5 to 14.8% across different hospital settings, with surgical wards reporting prevalence ranging from 5.7 to 45.8%. Among the reviewed studies, surgical site infection (SSI) was the most reported HCAI, with a prevalence ranging from 2.5 to 30.9%. Limited data on causative pathogens were available; however, a few studies have highlighted the significance of gram-negative rods, particularly in SSI and ventilator-associated pneumonia. Available data on endemic HCAI suggest that its prevalence is substantially higher than that in developed countries.

### Added value of this study

This systematic review and meta-analysis provide a comprehensive update on the magnitude and nature of HCAI in Africa, including data from 81,968 patients from 92 studies and 20 countries. We have summarised the recent epidemiology of endemic HCAI in Africa, examined differences in HCAI epidemiology between African Union regions and stratified our analysis by HCAI types. Further, we described bacterial aetiology and prevalence of HCAI associated AMR. By identifying the risk variables associated with HCAI, this review provides information that may be useful in supporting the design of focused and efficient infection prevention and control measures to reduce the burden of these infections in Africa.

### Implication of all the available evidence

Surveillance data play a key role in preventing HCAI as they enable healthcare facilities to track, monitor, and respond to trends, detect outbreaks early, identify risk factors, benchmark performance, and support quality improvement efforts. The heterogeneity in both the surveillance methods used and the prevalence of HCAI identified in our review suggests that existing definitions of HCAI types are not fit for purpose in the African context. Collection and analysis of timely and accurate surveillance data are essential for effective infection prevention and control programs. Pragmatic and appropriate HCAI definitions should be developed, validated, and implemented to both for local action and to estimate the burden of HCAI across Africa.

## Background

Healthcare-associated infection (HCAI) is a global health challenge that seriously threatens patient safety by significantly increasing the morbidity and mortality associated with healthcare exposure, hospital length of stay, long-term disability, financial burden, and contributing to the spread of multidrug-resistant (MDR) pathogens [1–6]. HCAI are thought to have a higher burden in African countries than in high-income countries, yet are understudied and underreported [7]. Data summarised in previous reviews report endemic HCAI prevalence of up to 15.5% in general wards and can reach 50% in intensive care units (ICU) in Africa [7–9]. However, it is important to emphasise that most of these studies focus on a particular institution, often tertiary and/or teaching facilities and may not reflect the situation at a national level.

Maintaining HCAI surveillance is a challenge in well-resourced healthcare settings [10] and even more so in low-resource ones, however defining the magnitude of HCAI is key to placing it in context for policy makers. It is anticipated that HCAI is responsible for a significant burden of disease in contexts where there is a lack of basic infection prevention and control (IPC) capacity. A further complication associated with HCAI is antimicrobial resistance (AMR), which has emerged as a major public health problem worldwide, with bacteria associated with HCAI disproportionately resistant to antibiotics [11, 12]. In low-resource settings, HCAI is likely to be exacerbated by infrastructural problems i.e. lack of water in hospitals (particularly safe water), poor hygiene and sanitation, understaffing, failure to implement or lack of antimicrobial policies, shortage of basic laboratory equipment for diagnosis, suboptimal adherence to safe practices by health care workers, limited compulsion to report HCAI, and limited funding [13, 14].

The World Health Organization (WHO) has developed several IPC guidelines and documents founded on the core components framework, a key aspect of which is HCAI surveillance [15, 16]. Data from HCAI surveillance can be used to quantify the HCAI burden, evaluate HCAI trends over time, pinpoint areas where HCAI prevention efforts need to be targeted and improved, and IPC strategies to reduce HCAI [15, 17].

Despite the significant impact of HCAI in Africa, there is a lack of up-to-date and comprehensive information on the prevalence, risk factors, and AMR of endemic HCAI in the region. This systematic review and meta-analysis of endemic HCAI in Africa is an update of the last 13 years since the last review published in 2011 by Nejad and colleagues [8], containing data published from 1995 to 2009. Here, we aimed to provide an up-to-date and comprehensive overview of the prevalence, risk factors, aetiology, and AMR of endemic HCAI in Africa.

## Methods

### Search strategy and eligibility criteria

For this systematic review and meta-analysis, we searched the MEDLINE/PubMed, CINAHL, and Global Health (EBSCOhost interface) electronic databases. To ensure literature saturation, the reference lists of the included studies were scanned to identify and capture other relevant studies, and Google Scholar was used to identify and screen studies citing them. Literature published in French and English between January 2010 and December 2022 was considered. The search was limited to human subjects and the most common HCAI encountered in African countries, including surgical site infections (SSI), healthcare-associated urinary tract infections (HA-UTI), healthcare-associated bloodstream infections (BSI), and hospital-acquired pneumonia/ventilator-associated pneumonia. The search strategy was developed based on the outcomes of interest (prevalence, risk factors, and antimicrobial resistance profile of bacteria isolated from HCAI). The complete search strategy with keywords and MeSH terms using the Boolean terms “OR” and “AND” is provided in supplemental materials (Supplement pp3-4).

We included observational studies (case-control, longitudinal, cohort, and cross-sectional) that prospectively or retrospectively explored the outcomes of interest (prevalence, risk factors, aetiologic agent and the antimicrobial resistance profile of bacteria isolated from HCAI) in all age groups in inpatient settings. We excluded the following types of studies due to the lack of relevance to our research question, or due to their limitations in allowing us to identify risk factors for HCAI or assess AMR: those reporting only the prevalence of HCAI without including at least one of the other outcomes of interest, those reporting on specific microorganisms causing HCAI, those reporting risk factors associated with HCAI but not reporting the effect size measures of these factors, or those reporting on HCAI outbreaks. Case series, case reports, editorials, commentaries, conference proceedings, preprints, reviews, previous systematic reviews and meta-analyses, and unpublished articles were excluded. Research letters to the editor containing data that met these criteria were included. We followed the Preferred Reporting Items for Systematic Review and Meta-Analysis 2020 (PRISMA) guidelines [18] (Supplement pp5-6) while conducting this systematic review and meta-analysis. The protocol was registered and published in PROSPERO (CRD42022374559).

### Data extraction

Two reviewers (GKB and EM) independently screened the titles and abstracts of studies according to the inclusion criteria. Any disagreements were resolved by discussion and consensus. In cases of further disagreement, a third reviewer (NF) holding a casting vote was consulted. The full-text review then occurred, and each reviewer independently screened the full texts against the inclusion and exclusion criteria, with disagreements resolved by consensus or discussion with a third reviewer (NF), if necessary.

Two reviewers (GKB and EM) extracted the data in a pre-piloted Excel spreadsheet. This included the study’s first author, publication year, region, country, population, study design, study population, surveillance definition used to define HCAI, type of HCAI and its prevalence, risk factors associated with HCAI (if analysed) and their effect size, and isolated bacteria and their antimicrobial resistance profile (if reported). The extracted data were compared, and discrepancies were resolved through discussion.

To assess the risk of bias in the included studies, we used a modified Critical Appraisal Skills Programme (CASP) checklist, designed to fit our research question and the Newcastle-Ottawa scale (NOS) for assessing the quality of non-randomized studies in meta-analyses (Supplement pp7-8) [19]. Both quality scales and domain-based tools were used simultaneously to assess all the included studies. The CASP checklist used in this systematic review assessed four domains: (i) appropriateness of the study population or participant recruitment, (ii) eligibility criteria, (iii) valid methods to identify the HCAI, and (iv) selective non-reporting or under-reporting of outcome measures. The NOS quality instrument score was awarded a star (corresponding to the points) for each area. It assesses the study’s area of selection (maximum of 5 points), comparability (maximum of 2 points), and outcomes (maximum of 3 points). After summing the star points, the studies were classified into three categories: good (7–10 points), moderate (5–6 points), and poor (0–4 points). The risk of bias assessment was performed by GKB and EM, and any disagreements were resolved by consensus.

### Data synthesis and statistical analysis

The extracted data were reported as study-level summary estimates, and qualitative and quantitative techniques were used to synthesise them. The primary outcome was the prevalence of HCAI stratified by HCAI type. Secondary outcomes included risk factors associated with HCAI and AMR profile of reported bacteria causing HCAI. All estimates were expressed as proportions with restricted maximum likelihood (REML) 95% confidence intervals (CI) and presented in forest plots using a random-effects meta-analysis. Heterogeneity was assessed using Higgins *I*^*2*^ statistic, Cochran’s Q test, and tau-squared τ^2^. The 95% CI around τ^2^ and *I*^*2*^ were calculated to assess confidence in these metrics. We set a stringent *I*^*2*^ threshold of > 75% as indicative of significant heterogeneity, but we also assessed this heterogeneity through the CIs and localisation on the forest plot [20]. Significant heterogeneity in prevalence between HCAI types was expected since some types of HCAI are more commonly reported, and this was addressed by stratification by HCAI type [8]. In the case of high heterogeneity, we determined it was not appropriate to pool estimates of HCAI prevalence [21].

To provide pooled risk estimates for the factors associated with HCAI, an exploratory meta-regression analysis was performed for risk factors found to be significant in at least four of the included studies. This threshold was chosen to ensure that the meta-regression analysis was based on an adequate number of studies to provide a robust and meaningful relationship between the pooled risk estimates and the outcome of interest. This approach helps reduce the risk of random chance or spurious associations and increases the validity of the meta-regression results. We presented the pooled estimated effect size (odds ratio (OR)) and degrees of heterogeneity with their 95% CI and p-values. The OR were computed and reported on a log scale. A statistically significant (*p* < 0.05) coefficient indicated an association between the effect estimate for HCAI and the associated risk factors.

We used quantile regression to calculate the AMR median rates and interquartile ranges (IQR) of the reported bacteria. We performed *post hoc* sensitivity analyses stratified by study quality to further estimate the robustness of the relevance estimates. All analyses were performed using the *meta* (version 6.1-0) and *metafor* (version 3.8-1) packages in R.

### Role of the funding sources

The funders of the study had no role in the study design, data collection, data analysis, data interpretation, or writing of the manuscript.

## Results

**Fig. 1:**
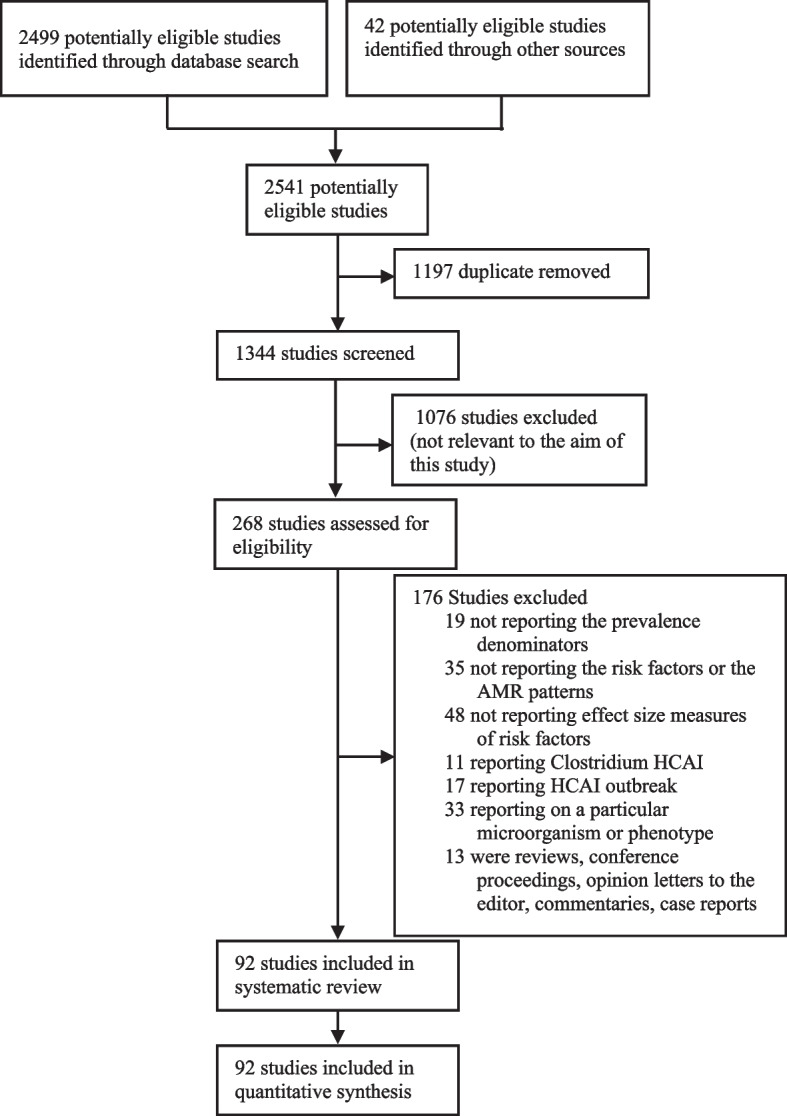
Flowchart of articles screening and selection

**Fig. 2:**
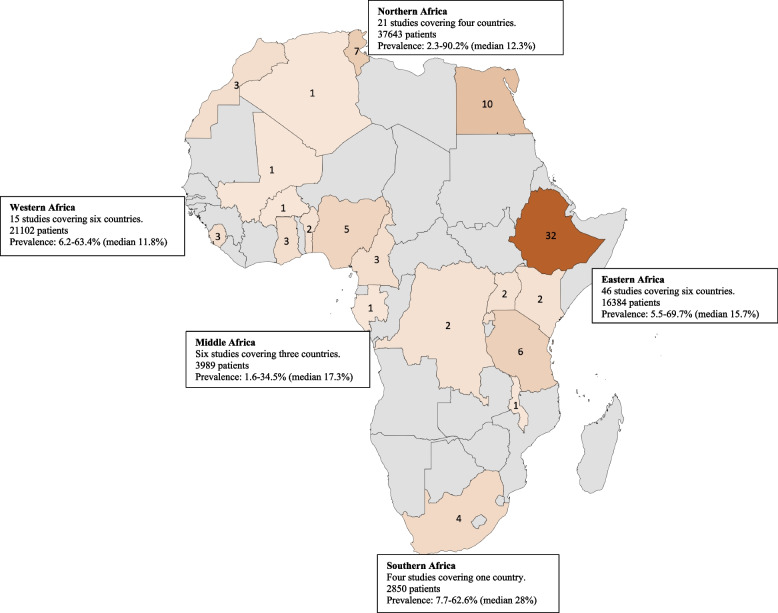
Geographical distribution and number of selected studies in different African countries

A total of 2541 studies were identified after a comprehensive search. Among them, 2499 studies were identified through the database we consulted and the remaining 42 were identified through other sources. Ninety-two studies from 20 African countries were included in the analysis (Fig. 1). The largest number of studies were from Ethiopia (*n* = 32, 34.8%) [22–53], Egypt (*n* = 10, 10.9%) [54–63], Tunisia (*n* = 7, 7.6%) [64–70], Tanzania (*n* = 6, 6.5%) [71–76], and Nigeria (*n* = 5, 5.4%) [77–81]. Other studies were from South Africa (*n* = 4, 4.3%) [82–85], Rwanda [86–88], Ghana [89–91], Cameroon [92–94], Sierra Leone [95–97], and Morocco [98–100] (for each, *n* = 3, 3.3%), Kenya [101, 102], Uganda [103, 104], Democratic Republic of Congo [105, 106], and Benin [107, 108] (for each, *n* = 2, 2.2%), Malawi [13], Gabon [109], Burkina Faso [110], Mali [111], and Algeria [112] (for each, *n* = 1, 1.1%). When classifying countries by UN African region, 46 (50%) studies were from Eastern Africa, 21 (22.8%) from Northern, 15 (16.3%) from Western, 6 (6.5%) from Middle, and 4 (4.4%) from Southern Africa (Fig. 2). These studies comprised a total of 81,968 patients. The sample size ranged from 32 to 15,502 patients per study. The geographical distribution of these patients by UN African region is as follow: 37,643 from Northern Africa, 21,102 from Western Africa, 16,384 from Eastern Africa, 3,989 from Middle Africa, and 2,850 from Southern Africa.

Most studies were conducted at university/teaching hospitals (*n* = 44, 47.8%) and other tertiary hospitals (*n* = 37, 40.2%). Approximately half of the included studies were conducted in the surgical (*n* = 31, 33.7%) and obstetrics wards (*n* = 18, 19.6%). Twenty (21.7%) studies were conducted in ICUs, while 16 (17.4%) reported HCAI from all wards of their hospitals. Most studies used the CDC HCAI surveillance definitions (*n* = 49, 53.3%), with a few examples of ECDC (*n* = 3, 3.3%) and WHO (*n* = 3, 3.3%) definitions being used. Two studies used both the CDC and ECDC definitions, while the IDSA and NINSS HCAI surveillance definitions were each used in one (1.1%) study. Among the studies that used CDC definitions, four adapted them to the local context. A total of 33 (35.9%) studies used their own surveillance definitions or did not report the definitions used in diagnosing HCAI. Study characteristics are summarised in Table 1.

**Table 1:** Characteristics of included studies

1st Authors, publication year	Region	Country	Type of setting	Study design/type	Targeted ward	Age group	Surveillance definition used	Population	HCAI Prevalence
Abdel-Wahab et al. 2013 [54]	Northern	Egypt	University/Teaching hospital	Prospective	Neonatal ICU	Neonates	Adapted CDC	238	21.4
Abosse et al. 2021 [22]	Eastern	Ethiopia	Tertiary hospital	Cross-sectional	All	All	NR	165	69.7
Abubakar, 2020 [77]	Western	Nigeria	University/Teaching hospital	PPS	All	All	ECDC	321	15.6
Ahoyo et al. 2014 [107]	Western	Benin	Tertiary hospital	PPS	All	All	CDC	3130	19.1
Akoko et al. 2012 [71]	Eastern	Tanzania	Tertiary hospital	Cross-sectional	Surgical	All	NINSS	118	35.6
Alemye et al. 2021 [23]	Eastern	Ethiopia	Tertiary hospital	Cross-sectional	Obstetrics	Women	CDC	1069	12.3
Atif et al. 2015 [112]	Northern	Algeria	University/Teaching hospital	Prospective	Surgical	Adults	NNIS^a^	593	5.4
Awoke et al. 2019 [34]	Eastern	Ethiopia	University/Teaching hospital	Cross-sectional	Surgical	All	NR	261	13.0
Ayala et al. 2021 [45]	Eastern	Ethiopia	University/Teaching hospital	Cross-sectional	Obstetrics	Women	NR	382	8.9
Ayed et al. 2019 [64]	Northern	Tunisia	University/Teaching hospital	PPS	All	All	CDC	752	10.9
Azeze et al. 2019 [48]	Eastern	Ethiopia	Tertiary hospital	Cross-sectional	Obstetrics	Women	NR	383	7.8
Bediako-Bowan et al. 2020 [89]	Western	Ghana	University/Teaching hospital	Cross-sectional	Surgical	Adults	CDC	3267	10.1
Bediako-Bowan et al. 2020 (2) [90]	Western	Ghana	University/Teaching hospital	Prospective	Surgical	All	CDC	358	16.2
Behari et al. 2015 [82]	Southern	South Africa	District hospital	Prospective	Surgical ICU	Adults	Own	32	25.0
Belay et al. 2022 [49]	Eastern	Ethiopia	Tertiary hospital	Retrospective	ICU	Adults	Own	312	27.9
Birhanu et al. 2022 [50]	Eastern	Ethiopia	University/Teaching hospital	Cross-sectional	Surgical	All	Own	373	19.3
Bizuayehu et al. 2022 [51]	Eastern	Ethiopia	Tertiary hospital	Cross-sectional	ICU	Adults	Own	220	51.4
Bizuayew et al. 2021 [52]	Eastern	Ethiopia	District hospital	Cross-sectional	Obstetrics	Women	CDC	622	12.4
Bunduki et al., 2021 [13]	Eastern	Malawi	University/Teaching hospital	PPS	Surgical	Adults	ECDC	105	11.4
Chernet et al. 2020 [55]	Northern	Egypt	University/Teaching hospital	Longitudinal	All	Adults	WHO	300	14.0
Dawit et al. 2021 [53]	Eastern	Ethiopia	Tertiary hospital	Longitudinal	ICU	Adults	NR	278	21.6
Dayyab et al. 2018 [81]	Western	Nigeria	Tertiary hospital	Prospective	Medical, Surgical, ICU, Dialysis unit	Adults	CDC	2334	6.2
De Nardo et al. 2016 [72]	Eastern	Tanzania	Tertiary hospital	Prospective cohort	Obstetrics	Women	CDC	467	48.2
Degbey et al. 2021 [108]	Western	Benin	University/Teaching hospital	Retrospective	Surgical	Adults	NR	384	7.8
Di Gennaro et al. 2020 [95]	Western	Sierra Leone	Tertiary hospital	Case–control	Obstetrics	Women	CDC	1016	25.0
Dramowski et al. 2015 [84]	Southern	South Africa	University/Teaching hospital	Retrospective	Neonatal ICU	Neonates	CDC	1145	62.6
Dramowski et al. 2016 [83]	Southern	South Africa	Tertiary hospital	Prospective	Paediatric ICU	Children	CDC/NHSN^a^	1347	31.0
Endalafer et al. 2011 [24]	Eastern	Ethiopia	University/Teaching hospital	Prospective	Surgical, ICU	Adults	CDC	215	39.1
Fisha et al. 2019 [25]	Eastern	Ethiopia	District hospital	Retrospective	Surgical	All	Own	642	10.0
Flouchi et al. 2022 [98]	Northern	Morocco	Tertiary hospital	Prospective	Surgical	All	CDC	2521	6.3
Gadallah et al. 2014 [56]	Northern	Egypt	University/Teaching hospital	Prospective	Neonatal ICU	Neonates	CDC	434	34.3
Galal et al. 2016 [57]	Northern	Egypt	University/Teaching hospital	Prospective	Paediatric ICU	Children	Own	427	30.9
Gelaw et al. 2017 [26]	Eastern	Ethiopia	General hospital	Cross-sectional	Obstetrics	Women	Own	384	6.8
Ghali et al. 2018 [65]	Northern	Tunisia	University/Teaching hospital	Prospective	Surgical	All	CDC	349	8.6
Gomaa et al. 2021 [58]	Northern	Egypt	Tertiary hospital	Case–control	Obstetrics	Women	NR	15,502	5.3
Hafez et al. 2012 [59]	Northern	Egypt	University/Teaching hospital	Prospective	Urology, Cardiothoracic	All	CDC	1062	17.6
Hajjej et al. 2014 [66]	Northern	Tunisia	Tertiary hospital	Prospective	Surgical ICU	Adults	IDSA	260	12.3
Halawi et al. 2018 [27]	Eastern	Ethiopia	University/Teaching hospital	Prospective	Surgical	All	Adapted CDC	131	20.6
Hassan et al. 2020 [60]	Northern	Egypt	Tertiary hospital	Prospective	All	All	NR	6912	3.7
Iwuafor et al. 2016 [78]	Western	Nigeria	University/Teaching hospital	Prospective cohort	ICU	Adults	Own	71	63.4
Jamoussi et al. 2018 [67]	Northern	Tunisia	University/Teaching hospital	PPS	ICU	Adults	Own	103	31.1
Kakupa et al. 2016 [105]	Middle	D.R. Congo	University/Teaching hospital	Cross-sectional	All	All	WHO	171	34.5
Kallel et al. 2010 [68]	Northern	Tunisia	University/Teaching hospital	Prospective cohort	ICU	Adults	CDC	261	21.1
Kefale et al. 2020 [28]	Eastern	Ethiopia	General hospital	Cross-sectional	Surgical	Adults	NR	281	19.6
Ketata et al. 2021 [69]	Northern	Tunisia	University/Teaching hospital	PPS	All	All	CDC/NHSN^a^	898	9.0
Ketema et al. 2020 [29]	Eastern	Ethiopia	Tertiary hospital	Prospective cohort	Obstetrics	Women	CDC	520	25.4
Kibwana et al. 2022 [73]	Eastern	Tanzania	University/Teaching hospital	Cross-sectional	Urology	All	NR	182	22.0
Kisibo et al. 2017 [74]	Eastern	Tanzania	Tertiary hospital	Cross-sectional	Orthopaedics	Adults	Own	300	24.0
Labi et al. 2019 [91]	Western	Ghana	Tertiary hospital	PPS	All	All	ECDC	2107	8.2
Lakoh et al. 2022 [96]	Western	Sierra Leone	Tertiary hospital	Prospective cohort	Surgical	Adults	CDC, ECDC	338	11.5
Lakoh et al. 2022 (2) [97]	Western	Sierra Leone	Tertiary hospital	Prospective	Surgical	Adults	CDC, ECDC	417	8.2
Laloto et al. 2017 [30]	Eastern	Ethiopia	University/Teaching hospital	Prospective	Surgical	All	CDC	105	19.0
Lijaemiro et al. 2020 [31]	Eastern	Ethiopia	Tertiary hospital	Prospective cohort	Obstetrics	Women	NR	166	15.1
Lubega et al. 2017 [104]	Eastern	Uganda	Tertiary hospital	Prospective	Surgical	All	Own	110	16.4
Lukuke et al. 2017 [106]	Middle	D.R. Congo	Tertiary hospital	Longitudinal	Obstetrics	Women	WHO	207	15.5
Mamo et al. 2017 [32]	Eastern	Ethiopia	University/Teaching hospital	Retrospective	Obstetrics	Women	CDC	384	9.4
Maoulainine et al. 2014 [99]	Northern	Morocco	Tertiary hospital	Prospective	Neonatal ICU	Neonates	CDC	702	13.0
Mawalla et al. 2011 [75]	Eastern	Tanzania	Tertiary hospital	Cross-sectional	Surgical	All	CDC	250	26.0
Melaku et al. 2012 [33]	Eastern	Ethiopia	Tertiary hospital	Cross-sectional	Surgical, gynaecology and obstetrics	Adults	CDC	1254	9.4
Mezemir et al. 2020 [35]	Eastern	Ethiopia	University/Teaching hospital	Cross-sectional	Surgical	All	CDC, NNIS^a^	249	24.5
Misha et al. 2021 [36]	Eastern	Ethiopia	University/Teaching hospital	Prospective	Surgical	Adults	NR	251	21.1
Misha et al. 2021 (2) [37]	Eastern	Ethiopia	University/Teaching hospital	Prospective cohort	Surgical	Adults	CDC	251	21.1
Mohamed et al. 2022 [38]	Eastern	Ethiopia	Tertiary hospital	Retrospective	Paediatric ICU	Children	NR	223	20.2
Molla et al. 2019 [39]	Eastern	Ethiopia	General hospital	Cross-sectional	Obstetrics	Women	CDC	334	8.1
Mpogoro et al. 2014 [76]	Eastern	Tanzania	Tertiary hospital	Prospective cohort	Obstetrics	Adults	CDC	312	10.9
Mukagendaneza et al. 2019 [86]	Eastern	Rwanda	University/Teaching hospital	Prospective	Surgical	Adults	CDC	294	11.6
Nair et al. 2018 [85]	Southern	South Africa	Tertiary hospital	PPS	All	All	CDC	326	7.7
Nanyunja et al. 2022 [103]	Eastern	Uganda	Tertiary hospital	Prospective cohort	Dialysis unit	Adults	Own	121	40.5
Nkurunziza et al. 2019 [87]	Eastern	Rwanda	District hospital	Prospective cohort	Obstetrics	Women	NR	550	10.9
Nouetchognou et al. 2016 [92]	Middle	Cameroon	University/Teaching hospital	Longitudinal	ICU, Gynaecology, Surgical, Neonatal unit	All	Own	307	19.2
Nwanko et al. 2016 [79]	Western	Nigeria	University/Teaching hospital	Prospective	Surgical	All	Own	5800	25.2
Olowo-okere et al. 2018 [80]	Western	Nigeria	University/Teaching hospital	Prospective cohort	Surgical	Adults, Children	CDC	135	25.9
Ouedraogo et al. 2020 [110]	Western	Burkina Faso	Tertiary hospital	Prospective	Surgical	All	CDC	964	11.8
Oumer et al. 2021 [40]	Eastern	Ethiopia	General hospital	Cross-sectional	All	Adults	CDC	231	16.9
Raouf et al. 2020 [61]	Northern	Egypt	Tertiary hospital	Prospective	Surgical	Adults	CDC/NHSN^a^	2510	2.3
Sahiledengle et al. 2020 [41]	Eastern	Ethiopia	University/Teaching hospital	Prospective	Paediatrics	Children	CDC/NHSN^a^	448	12.7
Saied et al. 2011 [62]	Northern	Egypt	University/Teaching hospital	Prospective	ICU	All	CDC	1575	38.1
Salem et al. 2011 [70]	Northern	Tunisia	University/Teaching hospital	PPS	All	All	CDC	1373	5.4
Sattar et al. 2018 [102]	Eastern	Kenya	University/Teaching hospital	Cross-sectional	ICU	All	Own	92	54.3
Scherbaum et al. 2014 [109]	Middle	Gabon	District hospital	Prospective	All	All	Adapted CDC	2925	1.6
See et al. 2013 [63]	Northern	Egypt	University/Teaching hospital	Prospective	ICU	All	Adapted CDC	523	90.2
Shakir et al. 2021 [42]	Eastern	Ethiopia	Tertiary hospital	Cross-sectional	Surgical	All	NR	306	11.8
Shimi et al. 2015 [100]	Northern	Morocco	University/Teaching hospital	Retrospective	ICU	All	CDC	535	11.2
Titus et al. 2021 [93]	Middle	Cameroon	Tertiary hospital	Cross-sectional	Surgical	Adults	NR	304	12.2
Togo et al. 2010 [111]	Western	Mali	University/Teaching hospital	Prospective	Surgical	All	CDC	460	9.6
Velin et al. 2021 [88]	Eastern	Rwanda	District hospital	Prospective	Obstetrics	Women	NR	795	5.7
Victor et al. 2013 [101]	Eastern	Kenya	University/Teaching hospital	Prospective	Surgical	Adults	CDC	262	6.9
Weldu et al. 2018 [43]	Eastern	Ethiopia	District hospital	Cross-sectional	Surgical	Adults	CDC	280	11.1
Wendmagegn et al. 2018 [44]	Eastern	Ethiopia	Tertiary hospital	Retrospective	Obstetrics	Women	Own	206	11.7
Wodajo et al. 2017 [46]	Eastern	Ethiopia	University/Teaching hospital	Cross-sectional	Obstetrics	Women	CDC	592	11.0
Yallew et al. 2016 [47]	Eastern	Ethiopia	University/Teaching hospital	Cross-sectional	All	All	CDC	908	14.9
Yaouba et al. 2016 [94]	Middle	Cameroon	Tertiary hospital	Prospective	Surgical, Gynaecology	Adults	CDC	75	30.7

*HCAI *Healthcare associated infection, *PPS* Point Prevalence Survey, *IDSA* Infectious Diseases Society of America, *ECDC* European Centre for Disease Prevention and Control, *CDC* Centers for Disease Control and Prevention, *NHSN* National Healthcare Safety Network, *NNIS* National Nosocomial Infections Surveillance System, *ICU* Intensive Care Unit, *NR* Not Reported, *WHO* World Health Organisation, *NINSS* National Integrated Non-Communicable Diseases Surveillance System

^a^Classified among the CDC definitions

The NOS and CASP risk bias assessment findings are detailed in the “Supplemental Materials” section (Supplement pp9-15). Of the 92 included studies, 13 (14.1%), 30 (32.6%), and 49 (53.3%) were assessed to be of poor, moderate, and good quality, respectively. The validity of the methods used to identify the HCAI, the sample size, and selective non-reporting or under-reporting of outcome measures were sources of bias.

HCAI prevalence was extremely heterogeneous across studies ranging from 1.6 to 90.2% with a median of 15% (Table 1). This heterogeneity was also observed when studies were reported by African Union regions: 5.5–69.7% (median 15.7%) for Eastern Africa, 2.3–90.2% (median 12.3%) for Northern Africa, 6.2–63.4% (median 11.8%) for Western Africa, 1.6–34.5% (median 17.3%) for Middle Africa, and 7.7–62.6% (median 28%) for Southern Africa (Fig. 2). When considering high quality studies, prevalence ranges varied from 2.4 to 85.7% (median 20.6%) for pneumonia, 2.4–41.2% (median 20.0%) for BSI, 0.5–28.6% (median 19.8%) for UTI, and 2.3–69.7% (median 12.4%) for SSI (Table 2). We performed *post-hoc* sensitivity testing by excluding studies of poor and moderate quality when computing the pooled prevalence. Prevalence ranges varied only slightly by HCAI types even amongst high quality studies. Pooled random-effect summary estimates were calculated using good quality studies and stratified by HCAI type to determine if this would explain the heterogeneity observed with the following results: pneumonia (29% [95% CI: 8–51%]), BSI (21% [95% CI: 14–28%]), UTI (17% [95% CI: 10–23%]), SSI (18% [95% CI: 14–21%]). Significant heterogeneity persisted in each stratum, as evidenced by *I*^*2*^ values ranging from 93 to 99% (Supplement pp16-17). There was not enough data to pool estimates for each HCAI syndrome by region.

**Table 2:** Prevalence ranges of HCAI syndromes in random effects model

*Type of HCAI*	*Prevalence ranges (%) (Median)*	*I*^*2*^* (%)*	*Tau-square*	*P-value*
Before sensitivity testing				
PN	2.4–85.7 (20.6)	98	0.0538	< 0.01
BSI	2.4–62.6 (19.8)	98	0.0192	< 0.01
UTI	0.5–65.6 (16.9)	97	0.0189	< 0.01
SSI	1.8–70.5 (13.6)	98	0.0204	0
Unknown^a^	1.6–63.4 (14.9)	99	0.0167	0
After 1st sensitivity testing^b^				
PN	2.4–85.7 (20.6)	97	0.0594	< 0.01
BSI	2.4–48.9 (19.5)	89	0.0123	< 0.01
UTI	0.5–51.4 (16.9)	97	0.0151	< 0.01
SSI	1.8–70.5 (11.7)	98	0.0213	0
Unknown^a^	3.9–63.4 (14.0)	98	0.0178	< 0.01
After 2nd sensitivity testing^a^				
PN	2.4–85.7 (20.6)	98	0.0831	< 0.01
BSI	2.4–41.2 (20.0)	93	0.0124	< 0.01
UTI	0.5–28.6 (19.8)	97	0.0094	< 0.01
SSI	2.3–69.7 (12.4)	97	0.0153	< 0.01
Unknown^a^	3.9–39.1 (13.5)	98	0.0154	< 0.01

*SSI* surgical site infection, *BSI* Bloodstream Infection, *UTI* Urinary Tract Infection, *PN* Pneumonia, *CI* confidence intervals

^a^Studies that reported all the four HCAI without giving prevalence for each type

^b^Excluding low quality studies

^c^Including only good quality studies

Numerous risk factors associated with HCAI in Africa have been reported (Supplement pp18-29). The exploratory meta-regression analysis identified different significant risk factors (Table 3). These included contaminated and dirty wounds (OR: 1.75, 95% CI: 1.31–2.19), hospital stay more than seven days (1.39, 0.92–1.80), presence of urinary catheter (1.57, 0.35–2.78), endotracheal intubation and mechanical ventilation (1.53, 0.85–2.22), and the presence of vascular catheter (1.49, 0.52–2.45). We did not perform meta-regression of risk factors by HCAI types because we did not have enough data.

**Table 3:** Exploratory Meta-regression analysis of risk factors associated with HCAI in Africa

Risk factors	Estimated effect size (OR)	95% CI	Std error	Z-value	*P*-value	*I*^*2*^ (%)	Q	*P*-value
Contaminated and dirty wound	1.75	1.31–2.19	0.223	7.84	< 0.001	0	11.33	0.88
Hospital stays (pre- or post-operative) > 7 days	1.36	0.92–1.80	0.225	6.02	< 0.0001	72.1	94.94	< 0.0001
Presence of urinary catheter	1.57	0.35–2.78	0.618	2.53	0.0114	73.8	29.4	< 0.0001
Endotracheal intubation and mechanical ventilation	1.53	0.85–2.22	0.350	4.41	< 0.0001	38.8	10.77	0.15
Presence of PVC or CVC	1.49	0.52–2.45	0.492	3.02	< 0.0025	62.3	16.14	0.013
Middle incision	1.49	0.66–2.32	0.430	3.50	0.0005	0	0.46	0.98
Haemoglobin level < 11 g/dl	1.42	0.78–2.05	0.322	4.39	< 0.0001	0	1.89	0.98
History of surgery in the past 30 days	1.26	0.76–1.76	0.256	4.92	< 0.0001	0	3.08	0.93
Blood transfusion	1.26	0.35–2.17	0.464	2.71	0.0067	40.9	9.41	0.09
Duration of procedure > 60 min	1.10	0.55–1.65	0.281	3.91	< 0.0001	2.83	12.43	0.26
Membrane rupture > 12 h	1.08	0.60–1.57	0.248	4.37	< 0.0001	28.6	10.33	0.28
Multiple vaginal examinations	1.02	0.23–1.81	0.402	2.53	0.0113	0	2.46	0.48
Duration of labour > 24 h	1.01	0.39–1.63	0.318	3.18	0.0015	0	3.16	0.79
Hypertension and hypertensive disorders	0.92	0.19–1.64	0.368	2.49	0.0128	0	2.64	0.62
Diabetes	0.88	0.38–1.39	0.256	3.42	< 0.0006	0	9.72	0.37

*PVC* peripheral venous catheter, *CVC* central venous catheter, *Std error* standard error, *CI* confidence intervals, *OR* odds ratio

Forty-eight studies reported microbiology results from HCAI. Overall, 6463 isolates were reported: 2145 (33.2%) from SSI, 1995 (30.8%) from BSI, 593 (9.2%) from UTI, and 527 (8.2%) from pneumonia (Table 4). The source of the remaining 1203 (18.6%) isolates was not identified. The most frequently reported bacteria causing HCAI were *E. coli* (18.3%, *n* = 1182), *S. aureus* (17.3%, *n* = 1118), *Klebsiella* spp. (17.2%, *n* = 1115), *Pseudomonas* spp. (10.3%, *n* = 671), and *Acinetobacter* spp. (6.8%, *n* = 438). The bacteria most isolated from SSI were *E. coli* (25.3%, 543/2145), *S. aureus* (20.6%, 443/2145), and *Klebsiella* spp. (11.8%, 252/2145). The microorganisms most commonly isolated from UTI were *E. coli* (24.8%, 147/593), *Candida* spp. (17.4%, 103/593), and *Klebsiella* spp. (13.8%, 82/593). The bacteria most frequently isolated from BSI were *Klebsiella* spp. (28.5%, 568/1995), CoNS (11.5%, 229/1995), *S. aureus* (11.4%, 227/1995), *Acinetobacter* spp. (10.0%, 200/1995), and *E. coli* (7.8%, 156/1995). The bacteria most frequently isolated from patients with pneumonia HCAI were *Acinetobacter* spp. (25.4%, 134/527), *Klebsiella* spp. (21.6%, 114/527), and *Pseudomonas* spp. (18.6%, 98/527) (Table 4).

**Table 4:** Bacteria reported in different HCAI clinical syndromes

Bacteria	SSI, n (%)	UTI^a^, n (%)	BSI^b^, n (%)	PN^c^, n (%)	Unknown^d^, n (%)	Total, n (%)
*Acinetobacter* spp*.*	44 (2.1)	26 (4.4)	200 (10.0)	134 (25.4)	34 (2.8)	438 (6.8)
*Citrobacter* spp*.*	67 (3.1)	6 (1.0)	6 (0.3)	.	45 (3.7)	124 (1.9)
CoNS	119 (5.5)	21 (3.5)	229 (11.5)	3 (0.6)	54 (4.5)	426 (6.6)
*E. coli*	543 (25.3)	147 (24.8)	156 (7.8)	40 (7.6)	296 (24.6)	1,182 (18.3)
*Enterobacter* spp*.*	23 (1.1)	16 (2.7)	68 (3.4)	16 (3.0)	32 (2.7)	155 (2.4)
*Enterococcus* spp*.*	92 (4.3)	52 (8.8)	117 (5.9)	6 (1.1)	107 (8.9)	374 (5.8)
*Klebsiella* spp*.*	252 (11.8)	82 (13.8)	568 (28.5)	114 (21.6)	99 (8.2)	1,115 (17.2)
*Kluyvera* spp*.*	5 (0.2)	.	.	.	.	5 (0.1)
*Morganella morganii*	5 (0.2)	1 (0.2)	3 (0.1)	1 (0.2)	.	10 (0.2)
*Proteus* spp*.*	251 (11.7)	25 (4.2)	3 (0.1)	15 (2.8)	22 (1.8)	316 (4.9)
*Pseudomonas* spp*.*	214 (10.0)	46 (7.8)	148 (7.4)	98 (18.6)	165 (13.7)	671 (10.3)
*S. aureus*	443 (20.6)	61 (10.3)	227 (11.4)	72 (13.7)	315 (26.2)	1,118 (17.3)
*Serratia* spp*.*	11 (0.5)	4 (0.7)	106 (5.3)	4 (0.8)	2 (0.2)	127 (2.0)
*Stenotrophomonas maltophilia*	1 (0.1)	.	11 (0.6)	.	15 (1.3)	27 (0.4)
*Streptococcus* spp*.*	62 (2.9)	3 (0.5)	78 (3.9)	9 (1.7)	1 (0.1)	153 (2.3)
*Haemophillus influenzae*	.	.	.	11 (2.1)	.	11 (0.2)
*Candida spp.*	13 (0.6)	103 (17.4)	75 (3.8)	4 (0.8)	16 (1.3)	211 (3.3)
**Total**	**2145**	**593**	**1995**	**527**	**1203**	**6463**

*SSI* surgical site infection, *BSI* Bloodstream Infection, *UTI* Urinary Tract Infection, *PN* Pneumonia

^a^Included catheter-associated UTI

^b^Included catheter-related BSI, central line-associated BSI

^c^Included healthcare-associated pneumonia, low respiratory tract infections, and ventilator-associated pneumonia

^d^Included all the four types of HCAI without separating them

Twenty studies reported bacterial antimicrobial susceptibility profiles. Bacteria commonly exhibited resistance to multiple antibiotics. A concerning level of resistance to third generation cephalosporins (70.3%, IQR: 50–100) was observed among Enterobacterales; 70.5% (IQR: 58.8–80.3) *S. aureus* were Methicillin Resistant; and 55% (IQR: 27.3–81.3) *Pseudomonas* spp. were resistant to all agents tested (Table 5). We did not perform AMR analysis for HCAI types nor by UN African region because most studies reported the overall AMR and not per HCAI types and because there were only 20 studies.

**Table 5:** Median resistance rates with interquartile ranges of selected bacteria to selected tested antibiotics

	Ciprofloxacin	Ceftriaxone	Ampicillin	Coamoxiclav	Gentamicin	Amikacin	Imipenem	Meropenem	Cotrimoxazole	Tetracycline	Chloramphenicol
*Acinetobacter* spp.	44 (16–72)	100 (36–100)	100 (99.2–100)	100 (75–100)	50 (11.4–75)	20 (10–72.4)	11 (0–30)	0 (0–33.3)	75 (28.6–80)	56 (50–80)	100 (44.4–100)
*Citrobacter* spp.	100 (45–100)	100 (25–100)	100 (81.8–100)	76 (45.5–100)	50 (26–100)	0 (0–25)	.	0 (0–45.5)	75 (25–100)	88 (72.7–100)	36 (27.3–100)
CoNS	27.3 (0–80)	.	20 (0–50)	60 (23–100)	13 (0–35)	.	.	.	75 (63.6–100)	55 (0–81.2)	32 (0–60)
*E. coli*	38 (22.3–77.8)	70.6 (64–100)	89 (77.3–100)	72.7 (28.6–85.7)	45 (31.4–66.7)	11.8 (0–19.4)	.	0 (0–16.7)	75 (57.1–100)	78 (58.3–87.5)	50 (28.6–66.7)
*Enterobacter* spp.	4.9 (0–100)	50 (50–90.2)	90.2 (85.7–100)	81 (50–100)	50 (0–87.8)	13 (0–25)	9.8 (9.8–13)	.	100 (80.5–100)	57.1 (50–57.1)	42.9 (0–50)
*Enterococcus* spp.	50 (50–60)	.	60 (40–72.2)	67 (23–67)	62.3 (50–100)	.	.	.	.	80 (61.1–83.3)	36 (18–50)
*Klebsiella* spp.	50 (14.3–81.8)	70 (61–94.6)	100 (91.6–100)	75 (60–100)	62.5 (50–75)	9.1 (0–50)	0 (0–9)	0 (0–11.8)	76 (62.5–90)	88 (50–89)	36 (29.4–67)
*Proteus* spp.	63 (25–100)	60 (50–64)	.	55.6 (50–73)	50 (22.2–50)	0 (0–63)	0 (0–38)	0 (0–33.3)	88 (0–100)	100 (77.8–100)	44.4 (44.4–100)
*Pseudomonas* spp.	30 (19.2–58.3)	60 (0–75)	100 (84.6–100)	100 (53.9–100)	50 (22–75)	24.6 (0–25)	16.7 (0–33.3)	23.1 (0–33.3)	88.5 (12.5–100)	74 (7.5–77.3)	34.6 (12.5–100)
*S. aureus*	33.3 (16.1–56)	70.5 (58.8–80.3)	71 (71–96)	81 (21.4–90)	33.3 (0–45)	.	.	.	71.4 (64–100)	38.7 (33.3–55)	36 (15–80)

*CoNS* Coagulase negative *S. aureus*, *Co-amoxiclav* Amoxicillin-clavulanic acid

## Discussion

The prevalence of HCAI in Africa is clearly high, and bacteria associated with infections are frequently antimicrobial-resistant. Available data also suggest that risk factors for HCAI in Africa are entirely predictable and can be mitigated through implementation of IPC programs and many tools to address the challenge of HCAI exist.

Overall, the HCAI prevalence ranged between 1.6 and 90.2% (median 15%). These rates are higher than pooled prevalence reported in Europe (6.5%) [6], Southeast Asia (9%) [113], the United States (4%) [114], Australia (9.9%) [115], and comparable to the pooled prevalence reported in a previous meta-analysis from developing countries (15.5%) [7]. The high prevalence of HCAI in Africa could be due to inadequate infection control and prevention measures which are often hindered by limited capacity for infection prevention and control, poor laboratory support, and limited funding [13]. Previous studies have demonstrated suboptimal adherence to hand hygiene protocols among healthcare workers in Africa that can be attributable to factors such as absence of safe water in healthcare facilities, inadequate healthcare built environment, inadequate knowledge and training, lack of personnel, and heavy workload [13, 116, 117].

Prevalence of HCAI did not vary much when analysed by HCAI types, with pneumonia, BSI, and UTI having medians of 20–21% and SSI 12%. These infections are largely associated with medical devices and can be prevented through appropriate infection prevention and control measures. Hand and environmental hygiene as well as injection safety practices should be promoted in African healthcare facilities. Contrary to previous studies that reported SSI as the most common HCAI in healthcare facilities in Africa, our study showed that SSI has a lower median prevalence than other HCAI, although high heterogeneity was present [8, 118]. After sensitivity testing that excluded studies with poor and moderate quality, this ranking remained the same. There is a need for more routine data to mitigate the impact of bias. Nevertheless, this prevalence is high and lack of adequate infection control before, during and after a surgical procedure coupled with non-compliant surgical antimicrobial prophylaxis are areas that can be addressed to reduce SSI in Africa [116].

Despite the increase in HCAI surveillance studies from the previous systematic review, overall quantity and quality of HCAI data remain poor. CDC and ECDC HCAI surveillance definitions are currently the most widely used, however, applying these definitions in African settings is typically difficult because they require diagnostic facilities such as microbiological laboratories and complex imaging (CT scan, MRI), which are frequently not available [8, 15, 119, 120]. Consequently, HCAI surveillance remains a significant challenge and the burden of HCAI is poorly described in Africa [3]. Comparing HCAI rates within and between countries is critical for raising awareness about HCAI and its prevention and control; however, it necessitates standardised approaches, including uniform definitions. The World Health Organisation (WHO) and US Centers for Disease Control and Prevention are actively addressing this issue by endeavouring to develop and validate a set of definitions and diagnostic criteria for different HCAI syndromes that will be useful in the absence of a full range of diagnostic microbiology or radiology facilities. The WHO has the authority to advocate for the adoption of standard definitions and this will facilitate the integration of African data into broader international datasets.

None of the risk factors for HCAI in Africa were a surprise and were consistent with global data [121]. Patients with multiple comorbidities or complicated chronic illnesses are more susceptible to frequent hospitalisation, which increases their risk of HCAI and colonisation or infection with multidrug-resistant pathogens. To mitigate the burden of HCAI, several strategies can be implemented [121]. There is already evidence globally, that interventions such as hand hygiene, environmental cleaning, surveillance, and multimodal approaches are cost-effective for the prevention and control of HCAI [122]. Overall, implementing a multimodal approach to HCAI prevention and control in Africa is necessary to reduce the burden of these infections and mitigate the risks to patient safety, and surveillance to quantify the problem and guide local action is key. Implementing a multimodal approach to IPC including enhancing healthcare worker training, implementing evidence-based IPC intervention bundles, and establishing effective surveillance systems sits at the heart of the WHO Core Components of IPC strategy and can be used to reduce the burden of these infections [15]. It is anticipated this holistic strategy will help to address the complex challenges associated with HCAI in Africa, promoting patient safety and contributing to the overall improvement of healthcare systems on the continent.

The most frequently reported bacteria were often resistant to multiple antimicrobial agents that are commonly available in most countries in Africa. These bacteria are typical nosocomial pathogens and among the priority AMR bacterial pathogens identified by WHO. Resistance to fluoroquinolones and β-lactam antibiotics (i.e. cephalosporins, penicillins, and carbapenems), which are typically first-line empirical therapy for severe infections poses a significant threat to patient safety, as AMR further complicates HCAI treatment.

Limitations of this study include the limited number of studies, especially of studies that reported bacterial aetiology and associated AMR profile. Most papers reported aggregate data that were not broken down by clinical speciality or hospital department or age. Further there was an absence of studies from the majority of African countries. Many studies have not described the effect size measures (OR, *p*-value, and confidence interval) of risk factors. In addition, most studies were conducted in teaching hospitals, with minimal data from district hospitals. Quality of the included studies was highly heterogeneous, however, we addressed this by sensitivity tests and ranking of reported HCAI rates was not affected. Lack of consistency in methods for assessing AMR make these data challenging to compare across studies, nor was there enough AMR data to report by HCAI types. This study did not report on the mortality due to HCAI as majority of included papers did not report this measure.

## Conclusions

Here, we provide a comprehensive review of the current HCAI situation in Africa. HCAI are a major problem in Africa, with a high prevalence, multiple risk factors, and increasing resistance to antimicrobial agents. It is imperative to build on this work by developing and validating HCAI definitions adapted to African settings and there is a pressing need to move HCAI surveillance beyond the realm of research studies and establish it as part of routine practice including in primary and secondary healthcare facilities. These measures are essential for policymakers to develop, evaluate, and improve appropriate HCAI prevention and control interventions to reduce the HCAI burden in Africa, AMR, and ultimately enhance the quality of healthcare in Africa.

### Supplementary Information

**Additional file 1.**

## Data Availability

All the data presented in this systematic review were obtained from published studies and are cited in the references. The protocol of this study was registered and available in PROSPERO (CRD42022374559). The manuscript of this paper is also available as Preprint at SSRN: https://ssrn.com/abstract=4499690 or 10.2139/ssrn.4499690.

## Abbreviations

HCAI: Healthcare-associated infections
OR: Odds ratio
AMR: Antimicrobial resistance
SSI: surgical site infection
MDR: multidrug-resistance
ICU: intensive care units
IPC: infection prevention and control
WHO: World Health Organization
HA-UTI: healthcare-associated urinary tract infections
BSI: bloodstream infections
PRISMA: Preferred Reporting Items for Systematic Review and Meta-Analysis
CASP: Critical Appraisal Skills Programme
NOS: Newcastle-Ottawa scale
REML: restricted maximum likelihood
CI: confidence intervals
PPS: Point Prevalence Survey
IDSA: Infectious Diseases Society of America
ECDC: European Centre for Disease Prevention and Control
CDC: Centers for Disease Control and Prevention
NHSN: National Healthcare Safety Network
NNIS: National Nosocomial Infections Surveillance System
NR: Not Reported
WHO: World Health Organisation
NINSS: National Integrated Non-Communicable Diseases Surveillance System
PVC: peripheral venous catheter
CVC: central venous catheter
Std error: standard error
CoNS: Coagulase negative *S. aureus*
Co-amoxiclav: Amoxicillin-clavulanic acid

## References

[1] Irek EOEO, Amupitan AA, Obadare TO, Aboderin AO (2018). A systematic review of healthcare-associated infections in Africa: an antimicrobial resistance perspective. Afr J Lab Med.

[2] Stewart S, Robertson C, Pan J, Kennedy S, Haahr L, Manoukian S (2021). Impact of healthcare-associated infection on length of stay. J Hosp Infect.

[3] WHO (2009). WHO guidelines on Hand Hygiene in Health Care. First Global Patient Safety Challenge Clean Care is Safer Care.

[4] Cassini A, Plachouras D, Eckmanns T, Abu Sin M, Blank HP, Ducomble T (2016). Burden of six Healthcare-Associated infections on European population health: estimating incidence-based disability-adjusted life years through a Population prevalence-based modelling study. PLoS Med..

[5] Guest JF, Keating T, Gould D, Wigglesworth N (2020). Modelling the annual NHS costs and outcomes attributable to healthcare-associated infections in England. BMJ Open..

[6] Suetens C, Latour K, Kärki T, Ricchizzi E, Kinross P, Moro ML (2018). Prevalence of healthcare-associated infections, estimated incidence and composite antimicrobial resistance index in acute care hospitals and long-term care facilities: results from two European point prevalence surveys, 2016 to 2017. Eurosurveillance.

[7] Allegranzi B, Nejad SB, Combescure C, Graafmans W, Attar H, Donaldson L (2011). Burden of endemic health-care-associated infection in developing countries: systematic review and meta-analysis. Lancet.

[8] Nejad SB, Allegranzi B, Syed SB, Ellis B, Pittet D (2011). Health-care-associated infection in Africa: a systematic review. Bull World Health Organ.

[9] Pittet D (2014). Burden of endemic healthcare-associated infection in Africa. 16th ICID abstracts. Int J Infect Dis.

[10] Russo PL, Shaban RZ, Macbeth D, Carter A, Mitchell BG (2018). Impact of electronic healthcare-associated infection surveillance software on infection prevention resources: a systematic review of the literature. J Hosp Infect.

[11] Richet HM, Mohammed J, McDonald LC, Jarvis WR (2001). Building communication networks: international network for the study and prevention of emerging antimicrobial resistance. Emerg Infect Dis.

[12] Burnham CAD, Leeds J, Nordmann P, O’Grady J, Patel J (2017). Diagnosing antimicrobial resistance. Nat Rev Microbiol.

[13] Bunduki GK, Feasey N, Henrion MYR, Noah P, Musaya J (2021). Healthcare-associated infections and antimicrobial use in surgical wards of a large urban central hospital in Blantyre, Malawi: a point prevalence survey. Infect Prev Pract.

[14] Kanyangarara M, Allen S, Jiwani SS, Fuente D (2021). Access to water, sanitation and hygiene services in health facilities in sub-Saharan Africa 2013–2018: results of health facility surveys and implications for COVID-19 transmission. BMC Health Serv Res.

[15] Storr J, Twyman A, Zingg W, Damani N, Kilpatrick C, Reilly J (2017). Core components for effective infection prevention and control programmes: new WHO evidence-based recommendations. Antimicrob Resist Infect Control.

[16] Tomczyk S, Twyman A, de Kraker MEA, Coutinho Rehse AP, Tartari E, Toledo JP (2022). The first WHO global survey on infection prevention and control in health-care facilities. Lancet Infect Dis.

[17] Allegranzi B, Kilpatrick C, Storr J, Kelley E, Park BJ, Donaldson L (2017). Global infection prevention and control priorities 2018–22: a call for action. Lancet Glob Heal.

[18] Page MJ, McKenzie JE, Bossuyt PM, Boutron I, Hoffmann TC, Mulrow CD (2021). The PRISMA 2020 statement: an updated guideline for reporting systematic reviews. BMJ.

[19] Stockdale AJ, Chaponda M, Beloukas A, Phillips RO, Matthews PC, Papadimitropoulos A (2017). Prevalence of hepatitis D virus infection in sub-saharan Africa: a systematic review and meta-analysis. Lancet Glob Heal.

[20] Higgins JPT, Thomas J, Chandler J, Cumpston M, Li T, Page MJ WV, editors. Cochrane Handbook for Systematic Reviews of Interventions version 6.3 (updated February 2022). Cochrane, 2022. Available from: www.training.cochrane.org/handbook.

[21] Munn Z, MClinSc SM, Lisy K, Riitano D, Tufanaru C (2015). Methodological guidance for systematic reviews of observational epidemiological studies reporting prevalence and cumulative incidence data. Int J Evid Based Healthc.

[22] Abosse S, Genet C, Derbie A (2021). Antimicrobial resistance profile of bacterial isolates identified from surgical site infections at a referral hospital, Northwest Ethiopia. Ethiop J Health Sci.

[23] Alemye T, Oljira L, Fekadu G, Mengesha MM (2021). Post cesarean section surgical site infection and associated factors among women who delivered in public hospitals in Harar city, Eastern Ethiopia: a hospital-based analytic cross-sectional study. PLoS ONE.

[24] Endalafer N, Gebre-Selassie S, Kotiso B (2011). Nosocomial bacterial infections in a tertiary hospital in Ethiopia. J Infect Prev.

[25] Fisha K, Azage M, Mulat G, Tamirat KS (2019). The prevalence and root causes of surgical site infections in public versus private hospitals in Ethiopia: a retrospective observational cohort study. Patient Saf Surg.

[26] Gelaw KA, Aweke AM, Astawesegn FH, Demissie BW, Zeleke LB (2017). Surgical site infection and its associated factors following cesarean section: a cross sectional study from a public hospital in Ethiopia. Patient Saf Surg.

[27] Halawi E, Assefa T, Hussen S (2018). Pattern of antibiotics use, incidence and predictors of surgical site infections in a Tertiary Care Teaching Hospital. BMC Res Notes.

[28] Kefale B, Tegegne GT, Degu A, Molla M, Kefale Y (2020). Surgical site infections and prophylaxis antibiotic use in the surgical ward of public hospital in western Ethiopia: a hospital-based retrospective cross-sectional study. Infect Drug Resist.

[29] Ketema DB, Wagnew F, Assemie MA, Ferede A, Alamneh AA, Leshargie CT (2020). Incidence and predictors of surgical site infection following cesarean section in North-West Ethiopia: a prospective cohort study. BMC Infect Dis.

[30] Laloto TL, Hiko Gemeda D, Abdella SH (2017). Incidence and predictors of surgical site infection in Ethiopia: prospective cohort. BMC Infect Dis.

[31] Lijaemiro H, Berhe Lemlem S, Tesfaye Deressa J (2020). Incidence of surgical site infection and factors associated among cesarean deliveries in selected government hospitals in Addis Ababa, Ethiopia, 2019. Obstet Gynecol Int.

[32] Mamo T, Abebe TW, Chichiabellu TY, Anjulo AA (2017). Risk factors for surgical site infections in obstetrics: a retrospective study in an Ethiopian referral hospital. Patient Saf Surg.

[33] Melaku S, Kibret M, Abera B, Gebre-Sellassie S (2012). Antibiogram of nosocomial urinary tract infections in Felege Hiwot referral hospital, Ethiopia. Afr Health Sci.

[34] Awoke N, Arba A, Girma A (2019). Magnitude of surgical site infection and its associated factors among patients who underwent a surgical procedure at Wolaita Sodo University Teaching and Referral Hospital, South Ethiopia. PLoS One.

[35] Mezemir R, Seid A, Gishu T, Demas T, Gize A (2020). Prevalence and root causes of surgical site infections at an academic trauma and burn center in Ethiopia: a cross-sectional study. Patient Saf Surg.

[36] Misha G, Chelkeba L, Melaku T (2021). Bacterial profile and antimicrobial susceptibility patterns of isolates among patients diagnosed with surgical site infection at a tertiary teaching hospital in Ethiopia: a prospective cohort study. Ann Clin Microbiol Antimicrob.

[37] Misha G, Chelkeba L, Melaku T (2021). Incidence, risk factors and outcomes of surgical site infections among patients admitted to Jimma Medical Center, South West Ethiopia: prospective cohort study. Ann Med Surg.

[38] Mohamed AA, Haftu H, Hadgu A, Seyoum D, Gebrekidan G, Ebrahim MM (2022). Prevalence, clinical profile and risk factors of nosocomial infection in Ayder Pediatric Intensive Care Unit, Tigray, Ethiopia. Int J Gen Med.

[39] Molla M, Temesgen K, Seyoum T, Melkamu M (2019). Surgical site infection and associated factors among women underwent cesarean delivery in Debretabor General Hospital, Northwest Ethiopia: hospital based cross sectional study. BMC Pregnancy Childbirth.

[40] Oumer Y, Dadi BR, Seid M, Biresaw G, Manilal A (2021). Catheter-associated urinary tract infection: incidence, associated factors and drug resistance patterns of bacterial isolates in southern Ethiopia. Infect Drug Resist.

[41] Sahiledengle B, Seyoum F, Abebe D, Geleta EN, Negash G, Kalu A (2020). Incidence and risk factors for hospital-acquired infection among paediatric patients in a teaching hospital: a prospective study in southeast Ethiopia. BMJ Open.

[42] Shakir A, Abate D, Tebeje F, Weledegebreal F (2021). Magnitude of surgical site infections, bacterial etiologies, associated factors and antimicrobial susceptibility patterns of isolates among post-operative patients in Harari region public hospitals, harar, eastern Ethiopia. Infect Drug Resist.

[43] Weldu MG, Berhane H, Berhe N, Haile K, Sibhatu Y, Gidey T (2018). Magnitude and determinant factors of surgical site infection in Suhul Hospital Tigrai, Northern Ethiopia: a cross-sectional study. Surg Infect (Larchmt).

[44] Wendmagegn TA, Abera GB, Tsehaye WT, Gebresslasie KB, Tella BG (2018). Magnitude and determinants of surgical site infecion among women underwent cesarean section in Ayder comprehensive specialized hospital Mekelle City, Tigray region, Northern Ethiopia, 2016. BMC Pregnancy Childbirth.

[45] Ayala D, Tolossa T, Markos J, Yilma MT (2021). Magnitude and factors associated with surgical site infection among mothers underwent cesarean delivery in Nekemte town public hospitals, western Ethiopia. PLoS One..

[46] Wodajo S, Belayneh M, Gebremedhin S (2017). Magnitude and factors associated with post-cesarean surgical site infection at Hawassa University teaching and referral hospital, Southern Ethiopia: a cross-sectional study. Ethiop J Health Sci.

[47] Yallew WW, Abera K, Feleke MY (2016). Point prevalence of hospital-acquired infections in two teaching hospitals of Amhara region in. Drug Healthc Patient Saf.

[48] Azeze GG, Bizuneh AD (2019). Surgical site infection and its associated factors following cesarean section in Ethiopia: a cross-sectional study. BMC Res Notes.

[49] Belay CM, Zewale TAb, Amlak BT, Abebe TG, Hailu G (2022). Incidence and predictors of ventilator-associated pneumonia among adult intubated patients in Bahir Dar Specialized Hospitals, 2021: a retrospective follow-up study. Int J Gen Med.

[50] Birhanu A, Amare HH, G/Mariam M, Girma T, Tadesse M, Assefa DG (2022). Magnitude of surgical site infection and determinant factors among postoperative patients, a cross sectional study. Ann Med Surg.

[51] Bizuayehu H, Bitew A, Abdeta A, Ebrahim S (2022). Catheter-associated urinary tract infections in adult intensive care units at a selected tertiary hospital, Addis Ababa, Ethiopia. PLoS One.

[52] Bizuayew H, Abebe H, Mullu G, Bewuket L, Tsega D, Alemye T (2021). Post-cesarean section surgical site infection and associated factors in East Gojjam Zone primary hospitals, Amhara region, North West Ethiopia, 2020. PLoS One.

[53] Dawit TC, Mengesha RE, Ebrahim MM, Tequare MH, Abraha HE (2021). Nosocomial sepsis and drug susceptibility pattern among patients admitted to adult intensive care unit of Ayder Comprehensive Specialized Hospital, Northern Ethiopia. BMC Infect Dis.

[54] Abdel-Wahab F, Ghoneim M, Khashaba M, El-Gilany AH, Abdel-Hady D (2013). Nosocomial infection surveillance in an Egyptian neonatal intensive care unit. J Hosp Infect.

[55] Chernet AZ, Dasta K, Belachew F, Zewdu B, Melese M, Ali MM (2020). Burden of healthcare-associated infections and associated risk factors at Adama hospital medical college, Adama, Oromia, Ethiopia. Drug Healthc Patient Saf.

[56] Gadallah MAH, Fotouh AMA, Habil IS, Imam SS, Wassef G (2014). Surveillance of health care-associated infections in a tertiary hospital neonatal intensive care unit in Egypt: 1-year follow-up. Am J Infect Control.

[57] Galal YS, Youssef MRL, Ibrahiem SK (2016). Ventilator-associated pneumonia: incidence, risk factors and outcome in paediatric intensive care units at Cairo university hospital. J Clin Diagnostic Res.

[58] Gomaa K, Abdelraheim AR, El Gelany S, Khalifa EM, Yousef AM, Hassan H (2021). Incidence, risk factors and management of post cesarean section surgical site infection (SSI) in a tertiary hospital in Egypt: a five year retrospective study. BMC Pregnancy Childbirth.

[59] Hafez S, Saied T, Hasan E, Elnawasany M, Ahmad E, Lloyd L (2012). Incidence and modifiable risk factors of surveillance of surgical site infections in Egypt: a prospective study. Am J Infect Control.

[60] Hassan R, El-Gilany AH, Abd elaal AM, El-Mashad N, Azim DA (2020). An overview of healthcare-associated infections in a tertiary care hospital in Egypt. Infect Prev Pract.

[61] Raouf M, Ghazal T, Kassem M, Agamya A, Amer A (2020). Surveillance of surgical-site infections and antimicrobial resistance patterns in a tertiary hospital in Alexandria, Egypt. J Infect Dev Ctries.

[62] Saied T, Elkholy A, Hafez SF, Basim H, Wasfy MO, El-Shoubary W (2011). Antimicrobial resistance in pathogens causing nosocomial bloodstream infections in university hospitals in Egypt. Am J Infect Control.

[63] See I, Lessa FC, ElAta OA, Hafez S, Samy K, El-Kholy A (2013). Incidence and pathogen distribution of healthcare-associated infections in pilot hospitals in Egypt. Infect Control Hosp Epidemiol.

[64] Ayed H, Ben, Yaich S, Trigui M, Jemaa M, Ben HM, Ben, Karray R (2019). Prevalence and risk factors of health care–associated infections in a limited resources country: a cross-sectional study. Am J Infect Control.

[65] Ghali H, Ben Rejeb M, Chahed C, Harrabi F, Ben Rejeb O, Ben Fredj S (2018). Incidence and risk factors of surgical site infection in general surgery department of a Tunisian tertiary teaching hospital: a prospective observational study. Can J Infect Control.

[66] Hajjej Z, Nasri M, Sellami W, Gharsallah H, Labben I, Ferjani M (2014). Incidence, risk factors and microbiology of central vascular catheterrelated bloodstream infection in an intensive care unit. J Infect Chemother.

[67] Jamoussi A, Ayed S, Ben Ismail K, Chtara K, Bouaziz M, Mokline A (2018). The prevalence of healthcare-associated infection in medical intensive care units in Tunisia. Results of the multi-centre nosorea1 study. Tunisie Medicale.

[68] Kallel H, Dammak H, Bahloul M, Ksibi H, Chelly H, Ben Hamida C (2010). Risk factors and outcomes of intensive care unit-acquired infections in a Tunisian ICU. Med Sci Monit.

[69] Ketata N, Ben Ayed H, Ben Hmida M, Trigui M, Ben Jemaa M, Yaich S (2021). Point prevalence survey of health-care associated infections and their risk factors in the tertiary-care referral hospitals of Southern Tunisia. Infect Dis Heal.

[70] Salem K, Ben MS, El, Letaief M, Bchir M, Soltani MS (2011). Epidemiological profile of health-care-associated infections in the central-east area of Tunisia. East Mediterr Heal J.

[71] Akoko LO, Mwanga AH, Fredrick F, Mbembati NM, Health C (2012). Risk factors of surgical site infection at Muhimbili National Hospital, Dar Es Salaam, Tanzania. East Cent African J Surg.

[72] De Nardo P, Gentilotti E, Nguhuni B, Vairo F, Chaula Z, Nicastri E (2016). Post-caesarean section surgical site infections at a Tanzanian tertiary hospital: a prospective observational study. J Hosp Infect.

[73] Kibwana UO, Manyahi J, Sensa V, Yongolo SC, Lyamuya E (2022). Predictors of surgical site infections among patients undergoing open urological surgery at a Tertiary Hospital, Tanzania: a cross sectional study. East Afr Heal Res J.

[74] Kisibo A, Ndume VA, Semiono A, Mika E, Sariah A, Protas J (2017). Surgical site infection among patients undergone orthopaedic surgery at Muhimbili Orthopaedic Institute, Dar Es Salaam, Tanzania. East Cent African J Surg.

[75] Mawalla B, Mshana SE, Chalya PL, Imirzalioglu C, Mahalu W (2011). Predictors of surgical site infections among patients undergoing major surgery at Bugando Medical Centre in Northwestern Tanzania. BMC Surg..

[76] Mpogoro FJ, Mshana SE, Mirambo MM, Kidenya BR, Gumodoka B, Imirzalioglu C (2014). Incidence and predictors of surgical site infections following caesarean sections at Bugando Medical Centre, Mwanza, Tanzania. Antimicrob Resist Infect Control.

[77] Abubakar U (2020). Point-prevalence survey of hospital acquired infections in three acute care hospitals in Northern Nigeria. Antimicrob Resist Infect Control.

[78] Iwuafor AA, Ogunsola FT, Oladele RO, Oduyebo OO, Desalu I, Egwuatu CC (2016). Incidence, clinical outcome and risk factors of intensive care unit infections in the Lagos university teaching hospital (LUTH), Lagos, Nigeria. Lazzeri C, editor. PLoS One.

[79] Nwankwo E, Edino S (2014). Seasonal variation and risk factors associated with surgical site infection rate in Kano, Nigeria. Turkish J Med Sci.

[80] Olowo-okere A, Ibrahim Y, Sani A, Olayinka B (2018). Occurrence of surgical site infections at a tertiary healthcare facility in Abuja, Nigeria: a prospective observational study. Med Sci.

[81] Dayyab FM, Iliyasu G, Aminu A, Habib ZG, Tiamiyu AB, Tambuwal SH (2018). A prospective study of hospital-acquired infections among adults in a tertiary hospital in north-western Nigeria. Trans R Soc Trop Med Hyg.

[82] Behari AA, Kalafatis N (2015). Incidence and outcome of ventilator-associated pneumonia in Inkosi Albert Luthuli and King Edward VIII hospital surgical intensive care units. South Afr J Crit Care.

[83] Dramowki A, Whitelaw A, Cotton MF (2016). Burden, spectrum, and impact of healthcare-associated infection at a South African children’s hospital. Ann Thorac Surg.

[84] Dramowski A, Madide A, Bekker A (2015). Neonatal nosocomial bloodstream infections at a referral hospital in a middle-income country: burden, pathogens, antimicrobial resistance and mortality. Paediatr Int Child Health.

[85] Nair A, Steinberg WJ, Habib T, Saeed H, Raubenheimer JE (2018). Prevalence of healthcare-associated infection at a tertiary hospital in the Northern Cape Province, South Africa. South Afr Fam Pract.

[86] Mukagendaneza MJ, Munyaneza E, Muhawenayo E, Nyirasebura D, Abahuje E, Nyirigira J (2019). Incidence, root causes, and outcomes of surgical site infections in a tertiary care hospital in Rwanda: a prospective observational cohort study. Patient Saf Surg.

[87] Nkurunziza T, Kateera F, Sonderman K, Gruendl M, Nihiwacu E, Ramadhan B (2019). Prevalence and predictors of surgical-site infection after caesarean section at a rural district hospital in Rwanda. Br J Surg.

[88] Velin L, Umutesi G, Riviello R, Muwanguzi M, Bebell LM, Yankurije M (2021). Surgical site infections and antimicrobial resistance after cesarean section delivery in rural Rwanda. Ann Glob Heal.

[89] Bediako-Bowan AAA, Mølbak K, Kurtzhals JAL, Owusu E, Debrah S, Newman MJ (2020). Risk factors for surgical site infections in abdominal surgeries in Ghana: emphasis on the impact of operating rooms door openings. Epidemiol Infect.

[90] Bediako-Bowan A, Owusu E, Debrah S, Kjerulf A, Newman MJ, Kurtzhals JALL (2020). Surveillance of surgical site infection in a teaching hospital in Ghana: a prospective cohort study. J Hosp Infect.

[91] Labi AK, Obeng-Nkrumah N, Owusu E, Bjerrum S, Bediako-Bowan A, Sunkwa-Mills G (2019). Multi-centre point-prevalence survey of hospital-acquired infections in Ghana. J Hosp Infect.

[92] Nouetchognou JS, Ateudjieu J, Jemea B, Mesumbe EN, Mbanya D (2016). Surveillance of nosocomial infections in the Yaounde University Teaching Hospital, Cameroon. BMC Res Notes.

[93] Ebogo Titus N, Nzinga J, Nchufor N, Njuma T, Ntih L, Sena G (2021). Epidemiology of surgical site infection following abdominal surgeries at a reference hospital in North-West Cameroon. J West African Coll Surg.

[94] Yaouba D, Ngaroua, Ngah JE, Perpoint T, Mbo Amvene J, Vanhems P (2016). Incidence and risk factors for surgical site infections in N’Gaoundéré Regional Hospital, Cameroon. Am J Infect Control..

[95] Di Gennaro F, Marotta C, Pisani L, Veronese N, Pisani V, Lippolis V (2020). Maternal caesarean section infection (MACSI) in Sierra Leone: a case-control study. Epidemiol Infect.

[96] Lakoh S, Yi L, Russell JBW, Zhang J, Sevalie S, Zhao Y (2022). The burden of surgical site infections and related antibiotic resistance in two geographic regions of Sierra Leone: a prospective study. Ther Adv Infect Dis.

[97] Lakoh S, Yi L, Sevalie S, Guo X, Adekanmbi O, Smalle IO (2022). Incidence and risk factors of surgical site infections and related antibiotic resistance in Freetown, Sierra Leone: a prospective cohort study. Antimicrob Resist Infect Control.

[98] Flouchi R, Far M, El, Hibatallah A, Elmniai A, Rhbibou I, Touzani I (2022). Incidence of surgical site infections and prediction of risk factors in a hospital center in Morocco. J Infect Dev Ctries.

[99] Maoulainine FMR, Elidrissi NS, Chkil G, Abba F, Soraa N, Chabaa L (2014). Épidémiologie De L’Infection Nosocomiale Bactérienne dans Un Service De Réanimation Néonatale Marocain. Arch Pediatr.

[100] Shimi A, Touzani S, Elbakouri N, Bechri B, Derkaoui A, Khatouf M (2015). Nosocomial pneumonia in ICU CHU Hassan II of Fez. Pan Afr Med J.

[101] Victor D, Gunturu R, Kariuki S, Hakeem A, Raja A, Kimang’a A (2013). Pattern of pathogens and their sensitivity isolated from surgical site infections at the Aga Khan University Hospital, Nairobi, Kenya. Ethiop J Health Sci.

[102] Sattar FAA, Quadros DRS, Olang P, Chokwe T (2018). Incidence of ventilator-associated pneumonia in the critical care unit at Kenyatta National Hospital, a public tertiary care hospital. East Afr Med J.

[103] Nanyunja D, Chothia M-Y, Opio KC, Ocama P, Bwanga F, Kiggundu D (2022). Incidence, microbiological aspects and associated risk factors of catheter-related bloodstream infections in adults on chronic haemodialysis at a tertiary hospital in Uganda. IJID Reg.

[104] Lubega A, Joel B, Justina Lucy N (2017). Incidence and etiology of surgical site infections among emergency postoperative patients in mbarara regional referral hospital, South Western Uganda. Surg Res Pract.

[105] Kakupa DK, Muenze PK, Byl B, Wilmet MD (2016). Etude de la prévalence des infections nosocomiales et des facteurs associes dans les deux hopitaux universitaires de Lubumbashi, République Démocratique Du Congo: Cas Des Cliniques Universitaires De Lubumbashi et l’Hôpital Janson Sendwe. Pan Afr Med J.

[106] Lukuke HM, Kasamba E, Mahuridi A, Ngatu NR, Narufumi S, Mukengeshayi AN (2017). L’incidence des infections nosocomiales urinaires et des sites opératoires dans la maternité de l’hôpital général de référence de katuba à lubumbashi en république Démocratique Du Congo. Pan Afr Med J.

[107] Ahoyo TA, Bankolé HS, Adéoti FM, Gbohoun AA, Assavèdo S, Amoussou-Guénou M (2014). Prevalence of nosocomial infections and anti-infective therapy in Benin: results of the first nationwide survey in 2012. Antimicrob Resist Infect Control..

[108] Dégbey C, Kpozehouen A, Coulibaly D, Chigblo P, Avakoudjo J, Ouendo EM (2021). Prevalence and factors associated with surgical site infections in the university clinics of traumatology and urology of the National University Hospital Centre Hubert Koutoukou Maga in Cotonou. Front Public Heal.

[109] Scherbaum M, Kösters K, Mürbeth RE, Ngoa UA, Kremsner PG, Lell B (2014). Incidence, pathogens and resistance patterns of nosocomial infections at a rural hospital in Gabon. BMC Infect Dis..

[110] Ouedraogo S, Kambire JL, Ouedraogo S, Ouangre E, Diallo I, Zida M (2020). Surgical site infection after digestive surgery: diagnosis and treatment in a context of limited resources. Surg Infect (Larchmt).

[111] Togo A, Traoré A, Kante L, Coulibaly Y, Diango D, Keita M, et al. Fighting Nosocomial Infection Rates in the General Surgery Department of the Teaching Hospital Gabriel Toure in Bamako, Mali. Open Biol J. 2010;3(3):87–91.

[112] Atif ML, Azouaou A, Bouadda N, Bezzaoucha A, Si-Ahmed M, Bellouni R (2015). Incidence and predictors of surgical site infection in a general surgery department in Algeria. Rev Epidemiol Sante Publique.

[113] Ling ML, Apisarnthanarak A, Madriaga G (2015). The burden of healthcare-associated infections in southeast Asia: a systematic literature review and meta-analysis. Clin Infect Dis.

[114] Magill SS, Edwards JR, Bamberg W, Beldavs ZG, Dumyati G, Kainer MA (2014). Multistate point-prevalence survey of health care–associated infections. N Engl J Med.

[115] Russo PL, Stewardson AJ, Cheng AC, Bucknall T, Mitchell BG (2019). The prevalence of healthcare associated infections among adult inpatients at nineteen large Australian acute-care public hospitals: a point prevalence survey. Antimicrob Resist Infect Control.

[116] Bunduki GK, Mukululi MP, Masumbuko CK, Uwonda SA (2020). Compliance of antibiotics used for surgical site infection prophylaxis among patients undergoing surgery in a Congolese teaching hospital. Infect Prev Pract.

[117] Kalata NL, Kamange L, Muula AS (2013). Adherence to hand hygiene protocol by clinicians and medical students at Queen Elizabeth Central Hospital, Blantyre-Malawi. Malawi Med J.

[118] Abubakar U, Amir O, Rodríguez-Baño J (2022). Healthcare-associated infections in Africa: a systematic review and meta-analysis of point prevalence studies. J Pharm Policy Pract.

[119] European Centre for Disease Prevention and Control (2016). Point prevalence survey of healthcare-associated infections and antimicrobial use in European acute care hospitals-protocol version 5.3.

[120] CDC NHSN, January. CDC / NHSN surveillance definitions for specific types of infections. 2021. Available at: https://www.cdc.gov/nhsn/pdfs/pscmanual/17pscnosinfdef_current.pdf.

[121] Ferreira E, Pina E, Sousa-Uva M, Sousa-Uva A (2017). Risk factors for health care–associated infections: from better knowledge to better prevention. Am J Infect Control.

[122] Rice S, Carr K, Sobiesuo P, Shabaninejad H, Orozco-Leal G, Kontogiannis V, et al. Economic evaluations of interventions to prevent and control health-care-associated infections: a systematic review. Lancet Infect Dis. 2023;3099(22). 10.1016/S1473-3099(22)00877-5. 10.1016/S1473-3099(22)00877-5 37001543

