# Supplementary material for: Prevalence, risk factors, and antimicrobial resistance of endemic healthcare-associated infections in Africa: a systematic review and meta-analysis
Source: BMC Infect Dis. 2024 Feb 2;24:158. doi: 10.1186/s12879-024-09038-0 (PMC10836007; doi:10.1186/s12879-024-09038-0)
Supplement: Supplementary file 1 — Additional file 1. [file 12879_2024_9038_MOESM1_ESM.docx]

**Supplemental Materials**

**Prevalence, risk factors, and antimicrobial resistance of endemic healthcare-associated infections in Africa: a systematic review and meta-analysis**

Gabriel Kambale Bunduki ^1,2,3*^, Effita Masoamphambe ^1,2^, Tilly Fox ^2^, Janelisa Musaya ^1^, Patrick Musicha ^1,2^, Nicholas Feasey ^1,2,4^

**^1^ Malawi-Liverpool-Wellcome Programme, Kamuzu University of Health Sciences, Malawi** (G K Bunduki MD, E Masoamphambe MSc, P Musicha PhD, Prof J Musaya PhD, Prof N Feasey, PhD)

**^2^ Department of Clinical Sciences, Liverpool School of Tropical Medicine, UK** (G K Bunduki MD; E Masoamphambe MSc, T Fox MSc, P Musicha PhD, Prof N Feasey PhD)

**^3^ Centre d’Excellence en Maladies Infectieuses et Soins Critiques du Graben (CEMISOCG), Faculty of Medicine, Université Catholique du Graben, DRC** (G K Bunduki MD)

**^4^ School of Medicine, University of St Andrews, St Andrews, UK** (Prof N Feasey, PhD)

***Correspondence to:**

Dr Gabriel Kambale Bunduki

Malawi Liverpool Wellcome Trust Research Programme

Blantyre

Malawi

[gbunduki@mlw.mw](mailto:gbunduki@mlw.mw)

#

Table of Contents

[Search strategy and selection criteria 3](#_Toc139316624)

[Table S1: Search results as of 14 January 2023 3](#_Toc139316625)

[Table S2: Preferred Reporting Items for Systematic Review and Meta-Analysis checklist 5](#_Toc139316626)

[HCAI in Africa: risk of bias tool using an adapted CASP 7](#_Toc139316627)

[Newcastle-Ottawa Scale for assessing the quality of non-randomized studies in meta-analyses 8](#_Toc139316628)

[Table S3: NOS assessment 9](#_Toc139316629)

[Table S4: CASP risk bias assessment 13](#_Toc139316630)

[Figure S1: Forest plot of good quality studies pooled by HCAI types 16](#_Toc139316631)

[Table S5: Significant risk factors reported in included studies 18](#_Toc139316632)

[Table S6: Pooled risk factors associated with HCAI in Africa 26](#_Toc139316633)

# Search strategy and selection criteria

From 2010 to 2022, all entries in MEDLINE/PubMed, CINAHAL, Global health (EBSCOhost interface) and Google Scholar, were searched for recent studies on HCAI, including the “burden”, or “epidemiology”, or “prevalence”, or “incidence”, or “magnitude”, or “risk factors”, or “antimicrobial resistance profile of bacteria” from HCAI. The main search terms are “nosocomial infection”, or “hospital-acquired infection” or “healthcare-associated infection” or “surgical site infection”, or “wound infection”, or “nosocomial tissue infection”, or “nosocomial soft-tissue infection”, or “hospital-acquired urinary tract infection”, or “catheter-associated urinary tract infection”, or “nosocomial urinary tract infection”, or “hospital-acquired urinary tract infection”, or “catheter-associated bloodstream infection”, or “central line bloodstream infection”, or “bloodstream infection”, or “nosocomial bacteraemia”, or “nosocomial bloodstream infection”, or “hospital-acquired bloodstream infection”, or “healthcare-associated septicaemia”, or “hospital-acquired septicaemia”, or “nosocomial septicaemia”, or “Ventilator-associated pneumonia”, or “hospital-acquired pneumonia”, or “healthcare-associated pneumonia”, or “device-associated pneumonia infection”, or “nosocomial low respiratory tract infection”, or “nosocomial pneumonia” in combination with  “sub-Saharan Africa”, or “Africa”, or “each individual country name”.

# Table S1: Search results as of 14 January 2023

| **#** | **Query** | **Results** | | |
| --- | --- | --- | --- | --- |
|  |  | **MEDLINE/PUBMED** | **CINAHL** | **GLOBAL HEALTH** |
| S1 | ( “burden”, or “epidemiology”, or “prevalence”, or “incidence”, or “magnitude”, or “risk factors”, or “antimicrobial resistance profile of bacteria” ) AND ( “nosocomial infection”, or “hospital-acquired infection” or “healthcare-associated infection” ) AND ( “sub-Saharan Africa”, or “Africa”, or “Burundi”, or “Cameroon”, or “Central African Republic”, or “Chad”, or “Republic of Congo”, or “DR Congo”, or “Equatorial Guinea”, or “Gabon”, or “São Tomé and Príncipe”, or “Comoros”, or “Djibouti”, or “Eritrea”, or “Ethiopia”, or “Kenya”, or “Madagascar”, or “Mauritius”, or “Rwanda”, or “Seychelles”, or “Somalia”, or “South Sudan”, or “Sudan”, or “Tanzania”, or “Uganda”, or “Algeria”, or “Egypt”, or “Libya”, or “Mauritania”, or “Morocco”, or “Sahrawi Republic”, or “Tunisia”, or “Angola”, or “Botswana”, or “Eswatini”, or “Lesotho”, or “Malawi”, or “Mozambique”, or “South Africa”, or “Zambia”, or “Zimbabwe”, or “Benin”, or “Burkina Faso”, or “Cabo Verde”, or “Cote d’Ivoire”, or “Gambia”, or “Ghana”, or “Guinea”, or “Guinea-Bissau”, or “Liberia”, or “Mali”, or “Niger”, or “Nigeria”, or “Senegal”, or “Sierra Leone”, or Togo” ) | 658 | 149 | 150 |
| S2 | ( “burden”, or “epidemiology”, or “prevalence”, or “incidence”, or “magnitude”, or “risk factors”, or “antimicrobial resistance profile of bacteria” ) AND ( “surgical site infection”, or “wound infection”, or “nosocomial tissue infection”, or nosocomial soft-tissue infection” ) AND ( “sub-Saharan Africa”, or “Africa”, or “Burundi”, or “Cameroon”, or “Central African Republic”, or “Chad”, or “Republic of Congo”, or “DR Congo”, or “Equatorial Guinea”, or “Gabon”, or “São Tomé and Príncipe”, or “Comoros”, or “Djibouti”, or “Eritrea”, or “Ethiopia”, or “Kenya”, or “Madagascar”, or “Mauritius”, or “Rwanda”, or “Seychelles”, or “Somalia”, or “South Sudan”, or “Sudan”, or “Tanzania”, or “Uganda”, or “Algeria”, or “Egypt”, or “Libya”, or “Mauritania”, or “Morocco”, or “Sahrawi Republic”, or “Tunisia”, or “Angola”, or “Botswana”, or “Eswatini”, or “Lesotho”, or “Malawi”, or “Mozambique”, or “South Africa”, or “Zambia”, or “Zimbabwe”, or “Benin”, or “Burkina Faso”, or “Cabo Verde”, or “Cote d’Ivoire”, or “Gambia”, or “Ghana”, or “Guinea”, or “Guinea-Bissau”, or “Liberia”, or “Mali”, or “Niger”, or “Nigeria”, or “Senegal”, or “Sierra Leone”, or Togo” ) | 643 | 146 | 199 |
| S3 | ( “burden”, or “epidemiology”, or “prevalence”, or “incidence”, or “magnitude”, or “risk factors”, or “antimicrobial resistance profile of bacteria” ) AND ( “hospital-acquired urinary tract infection”, or “catheter-associated urinary tract infection”, or “nosocomial urinary tract infection”, or “hospital-acquired urinary tract infection” ) AND ( “sub-Saharan Africa”, or “Africa”, or “Burundi”, or “Cameroon”, or “Central African Republic”, or “Chad”, or “Republic of Congo”, or “DR Congo”, or “Equatorial Guinea”, or “Gabon”, or “São Tomé and Príncipe”, or “Comoros”, or “Djibouti”, or “Eritrea”, or “Ethiopia”, or “Kenya”, or “Madagascar”, or “Mauritius”, or “Rwanda”, or “Seychelles”, or “Somalia”, or “South Sudan”, or “Sudan”, or “Tanzania”, or “Uganda”, or “Algeria”, or “Egypt”, or “Libya”, or “Mauritania”, or “Morocco”, or “Sahrawi Republic”, or “Tunisia”, or “Angola”, or “Botswana”, or “Eswatini”, or “Lesotho”, or “Malawi”, or “Mozambique”, or “South Africa”, or “Zambia”, or “Zimbabwe”, or “Benin”, or “Burkina Faso”, or “Cabo Verde”, or “Cote d’Ivoire”, or “Gambia”, or “Ghana”, or “Guinea”, or “Guinea-Bissau”, or “Liberia”, or “Mali”, or “Niger”, or “Nigeria”, or “Senegal”, or “Sierra Leone”, or Togo” ) | 27 | 8 | 15 |
| S4 | ( “burden”, or “epidemiology”, or “prevalence”, or “incidence”, or “magnitude”, or “risk factors”, or “antimicrobial resistance profile of bacteria” ) AND ( “catheter-associated bloodstream infection”, or “central line bloodstream infection”, or “bloodstream infection”, or “nosocomial bacteraemia”, or “nosocomial bloodstream infection”, or “hospital-acquired bloodstream infection”, or “healthcare-associated septicaemia”, or “hospital-acquired septicaemia”, or “nosocomial septicaemia” ) AND ( “sub-Saharan Africa”, or “Africa”, or “Burundi”, or “Cameroon”, or “Central African Republic”, or “Chad”, or “Republic of Congo”, or “DR Congo”, or “Equatorial Guinea”, or “Gabon”, or “São Tomé and Príncipe”, or “Comoros”, or “Djibouti”, or “Eritrea”, or “Ethiopia”, or “Kenya”, or “Madagascar”, or “Mauritius”, or “Rwanda”, or “Seychelles”, or “Somalia”, or “South Sudan”, or “Sudan”, or “Tanzania”, or “Uganda”, or “Algeria”, or “Egypt”, or “Libya”, or “Mauritania”, or “Morocco”, or “Sahrawi Republic”, or “Tunisia”, or “Angola”, or “Botswana”, or “Eswatini”, or “Lesotho”, or “Malawi”, or “Mozambique”, or “South Africa”, or “Zambia”, or “Zimbabwe”, or “Benin”, or “Burkina Faso”, or “Cabo Verde”, or “Cote d’Ivoire”, or “Gambia”, or “Ghana”, or “Guinea”, or “Guinea-Bissau”, or “Liberia”, or “Mali”, or “Niger”, or “Nigeria”, or “Senegal”, or “Sierra Leone”, or Togo” ) | 180 | 45 | 103 |
| S5 | ( “burden”, or “epidemiology”, or “prevalence”, or “incidence”, or “magnitude”, or “risk factors”, or “antimicrobial resistance profile of bacteria” ) AND ( “Ventilator-associated pneumonia”, or “hospital-acquired pneumonia”, or “healthcare-associated pneumonia”, or “device-associated pneumonia infection”, or “nosocomial low respiratory tract infection”, or “nosocomial pneumonia” ) AND ( “sub-Saharan Africa”, or “Africa”, or “Burundi”, or “Cameroon”, or “Central African Republic”, or “Chad”, or “Republic of Congo”, or “DR Congo”, or “Equatorial Guinea”, or “Gabon”, or “São Tomé and Príncipe”, or “Comoros”, or “Djibouti”, or “Eritrea”, or “Ethiopia”, or “Kenya”, or “Madagascar”, or “Mauritius”, or “Rwanda”, or “Seychelles”, or “Somalia”, or “South Sudan”, or “Sudan”, or “Tanzania”, or “Uganda”, or “Algeria”, or “Egypt”, or “Libya”, or “Mauritania”, or “Morocco”, or “Sahrawi Republic”, or “Tunisia”, or “Angola”, or “Botswana”, or “Eswatini”, or “Lesotho”, or “Malawi”, or “Mozambique”, or “South Africa”, or “Zambia”, or “Zimbabwe”, or “Benin”, or “Burkina Faso”, or “Cabo Verde”, or “Cote d’Ivoire”, or “Gambia”, or “Ghana”, or “Guinea”, or “Guinea-Bissau”, or “Liberia”, or “Mali”, or “Niger”, or “Nigeria”, or “Senegal”, or “Sierra Leone”, or Togo” ) | 103 | 22 | 51 |

# Table S2: Preferred Reporting Items for Systematic Review and Meta-Analysis checklist

| **Section and Topic** | **Item #** | **Checklist item** | **Location where item is reported** |
| --- | --- | --- | --- |
| **TITLE** | | |  |
| Title | 1 | Identify the report as a systematic review. | Pg1 |
| **ABSTRACT** | | |  |
| Abstract | 2 | See the PRISMA 2020 for Abstracts checklist. | Pg2 |
| **INTRODUCTION** | | |  |
| Rationale | 3 | Describe the rationale for the review in the context of existing knowledge. | Pg6-7 |
| Objectives | 4 | Provide an explicit statement of the objective(s) or question(s) the review addresses. | Pg7 |
| **METHODS** | | |  |
| Eligibility criteria | 5 | Specify the inclusion and exclusion criteria for the review and how studies were grouped for the syntheses. | Pg7 |
| Information sources | 6 | Specify all databases, registers, websites, organisations, reference lists and other sources searched or consulted to identify studies. Specify the date when each source was last searched or consulted. | Pg7 |
| Search strategy | 7 | Present the full search strategies for all databases, registers and websites, including any filters and limits used. | Pg7 |
| Selection process | 8 | Specify the methods used to decide whether a study met the inclusion criteria of the review, including how many reviewers screened each record and each report retrieved, whether they worked independently, and if applicable, details of automation tools used in the process. | Pg8 |
| Data collection process | 9 | Specify the methods used to collect data from reports, including how many reviewers collected data from each report, whether they worked independently, any processes for obtaining or confirming data from study investigators, and if applicable, details of automation tools used in the process. | Pg8 |
| Data items | 10a | List and define all outcomes for which data were sought. Specify whether all results that were compatible with each outcome domain in each study were sought (e.g. for all measures, time points, analyses), and if not, the methods used to decide which results to collect. | Pg8 |
|  | 10b | List and define all other variables for which data were sought (e.g. participant and intervention characteristics, funding sources). Describe any assumptions made about any missing or unclear information. | Pg8 |
| Study risk of bias assessment | 11 | Specify the methods used to assess risk of bias in the included studies, including details of the tool(s) used, how many reviewers assessed each study and whether they worked independently, and if applicable, details of automation tools used in the process. | Pg8 |
| Effect measures | 12 | Specify for each outcome the effect measure(s) (e.g. risk ratio, mean difference) used in the synthesis or presentation of results. | Pg9 |
| Synthesis methods | 13a | Describe the processes used to decide which studies were eligible for each synthesis (e.g. tabulating the study intervention characteristics and comparing against the planned groups for each synthesis (item #5)). | Pg7-8 |
|  | 13b | Describe any methods required to prepare the data for presentation or synthesis, such as handling of missing summary statistics, or data conversions. | Pg7-8 |
|  | 13c | Describe any methods used to tabulate or visually display results of individual studies and syntheses. | Pg9 |
|  | 13d | Describe any methods used to synthesize results and provide a rationale for the choice(s). If meta-analysis was performed, describe the model(s), method(s) to identify the presence and extent of statistical heterogeneity, and software package(s) used. | Pg9 |
|  | 13e | Describe any methods used to explore possible causes of heterogeneity among study results (e.g. subgroup analysis, meta-regression). | Pg9-10 |
|  | 13f | Describe any sensitivity analyses conducted to assess robustness of the synthesized results. | Pg10 |
| Reporting bias assessment | 14 | Describe any methods used to assess risk of bias due to missing results in a synthesis (arising from reporting biases). | Pg9 |
| Certainty assessment | 15 | Describe any methods used to assess certainty (or confidence) in the body of evidence for an outcome. | Pg9 |
| **RESULTS** | | |  |
| Study selection | 16a | Describe the results of the search and selection process, from the number of records identified in the search to the number of studies included in the review, ideally using a flow diagram. | Pg11 |
|  | 16b | Cite studies that might appear to meet the inclusion criteria, but which were excluded, and explain why they were excluded. | Pg11 |
| Study characteristics | 17 | Cite each included study and present its characteristics. | Pg11 |
| Risk of bias in studies | 18 | Present assessments of risk of bias for each included study. | Pg12 |
| Results of individual studies | 19 | For all outcomes, present, for each study: (a) summary statistics for each group (where appropriate) and (b) an effect estimate and its precision (e.g. confidence/credible interval), ideally using structured tables or plots. | Pg11 |
| Results of syntheses | 20a | For each synthesis, briefly summarise the characteristics and risk of bias among contributing studies. | Pg12 |
|  | 20b | Present results of all statistical syntheses conducted. If meta-analysis was done, present for each the summary estimate and its precision (e.g. confidence/credible interval) and measures of statistical heterogeneity. If comparing groups, describe the direction of the effect. | Pg12 |
|  | 20c | Present results of all investigations of possible causes of heterogeneity among study results. | Pg12 |
|  | 20d | Present results of all sensitivity analyses conducted to assess the robustness of the synthesized results. | Pg12 |
| Reporting biases | 21 | Present assessments of risk of bias due to missing results (arising from reporting biases) for each synthesis assessed. | Pg12 |
| Certainty of evidence | 22 | Present assessments of certainty (or confidence) in the body of evidence for each outcome assessed. | Pg12 |
| **DISCUSSION** | | |  |
| Discussion | 23a | Provide a general interpretation of the results in the context of other evidence. | Pg14 |
|  | 23b | Discuss any limitations of the evidence included in the review. | Pg16 |
|  | 23c | Discuss any limitations of the review processes used. | Pg16 |
|  | 23d | Discuss implications of the results for practice, policy, and future research. | Pg15-16 |
| **OTHER INFORMATION** | | |  |
| Registration and protocol | 24a | Provide registration information for the review, including register name and registration number, or state that the review was not registered. | Pg8 |
|  | 24b | Indicate where the review protocol can be accessed, or state that a protocol was not prepared. | Pg8 |
|  | 24c | Describe and explain any amendments to information provided at registration or in the protocol. | Pg8 |
| Support | 25 | Describe sources of financial or non-financial support for the review, and the role of the funders or sponsors in the review. | Pg17 |
| Competing interests | 26 | Declare any competing interests of review authors. | Pg17 |
| Availability of data, code and other materials | 27 | Report which of the following are publicly available and where they can be found: template data collection forms; data extracted from included studies; data used for all analyses; analytic code; any other materials used in the review. | Pg17 |

# HCAI in Africa: risk of bias tool using an adapted CASP

Adapted from CASP checklists, and: Development of a quality appraisal tool for case series using a modified Delphi technique (Institute of Health Economics) 2012 (https://casp.uk.net accessed 14th February 2023 and Stockdale et al Lancet Global Health 2017 5(10):PE992-E1003)

**Domain 1: Study population/participant recruitment appropriateness**

Are the characteristics of the participants included in the study adequately described?

**Yes**: The authors should report the total number, age, and gender distribution of the participants who had HCAI

**Partially reported**: The criteria above are incompletely reported

**No**: None of the relevant characteristics of the participants is reported

**Domain 2. Are the eligibility criteria (inclusion and exclusion criteria) to enter the study explicit and appropriate?**

**Yes**: The eligibility criteria are clearly stated and replicable.

**Partially reported**: The criteria above are incompletely reported

**No**: The eligibility criteria are not clearly stated or are inappropriate.

**Domain 3: Did the study use valid methods to identify the HCAI?**

**Yes**: The CDC or ECD method was used

**Partially**: Adapted CDC or ECDC or any other definition from a recognised organisation was (for instance WHO, IDSA) used

**No**: The authors used their own definitions, or they did not report the definition they used.

**Domain 4: Selective non-reporting or under-reporting of the outcome measures**

**Yes**: Both Risk factors and AMR were selectively not reported or under-reported

**Partially**: One of the above was selectively not reported or under-reported

**No**: None

# Newcastle-Ottawa Scale for assessing the quality of non-randomized studies in meta-analyses

**Selection:**

1. Representativeness of the sample:
   1. Truly representative of the average in the target population. * (all subjects or random sampling)
   2. Somewhat representative of the average in the target group. * (non-random sampling)
   3. Selected group of users/convenience sample.
   4. No description of the derivation of the included subjects.
2. Sample size:
   1. Justified and satisfactory (including sample size calculation). *
   2. Not justified.
   3. No information provided
3. Non-respondents:
   1. Proportion of target sample recruited attains pre-specified target or basic summary of non-respondent characteristics in sampling frame recorded. *
   2. Unsatisfactory recruitment rate, no summary data on non-respondents.
   3. No information provided
4. Ascertainment of the exposure (risk factor):
   1. Vaccine records/vaccine registry/clinic registers/hospital records only. **
   2. Parental or personal recall and vaccine/hospital records. *
   3. Parental/personal recall only.

**Comparability:** (Maximum 2 stars)

1. Comparability of subjects in different outcome groups on the basis of design or analysis. Confounding factors controlled.
   1. Data/ results adjusted for relevant predictors/risk factors/confounders e.g. age, sex, time since vaccination, etc. **
   2. Data/results not adjusted for all relevant confounders/risk factors/information not provided.

**Outcome:**

1. Assessment of outcome:
   1. Independent blind assessment using objective validated laboratory methods. **
   2. Unblinded assessment using objective validated laboratory methods. **
   3. Used non-standard or non-validated laboratory methods with gold standard. *
   4. No description/non-standard laboratory methods used.
2. Statistical test:
   1. Statistical test used to analyse the data clearly described, appropriate and measures of association presented including confidence intervals and probability level (p value). *
   2. Statistical test not appropriate, not described or incomplete.

**Risk bias assessment scale**

**Good: 7-10 points**

**Moderate: 5-6 points**

**Poor: 0 to 4 points**

# Table S3: NOS assessment

| **Authors** | Representatives of sample | Sample size | Non-respondents | Ascertainment of exposure | Comparability based on design and analysis | Assessment of outcomes | Statistical test | Quality score | Quality scale |
| --- | --- | --- | --- | --- | --- | --- | --- | --- | --- |
| Abdel-Wahab et al., 2013 | 0 | 0 | N/A | 2 | 2 | 2 | 1 | 7 | Good |
| Abosse et al., 2021 | 1 | 1 | N/A | 2 | 2 | 2 | 1 | 9 | Good |
| Abubakar, 2020 | 1 | 0 | N/A | 2 | 2 | 0 | 1 | 6 | Moderate |
| Ahoyo et al., 2014 | 1 | 0 | N/A | 0 | 2 | 2 | 1 | 6 | Moderate |
| Akoko et al., 2012 | 1 | 0 | N/A | 2 | 2 | 0 | 1 | 6 | Moderate |
| Alemye et al., 2021 | 1 | 1 | N/A | 2 | 2 | 0 | 1 | 7 | Good |
| Atif et al., 2015 | 1 | 0 | N/A | 2 | 2 | 0 | 1 | 6 | Moderate |
| Awoke et al., 2019 | 1 | 1 | N/A | 2 | 2 | 0 | 1 | 7 | Good |
| Ayala et al., 2021 | 1 | 1 | N/A | 2 | 2 | 0 | 1 | 7 | Good |
| Ayed et al., 2019 | 1 | 0 | N/A | 2 | 2 | 0 | 1 | 6 | Moderate |
| Azeze et al., 2019 | 1 | 1 | N/A | 2 | 2 | 0 | 1 | 7 | Good |
| Bediako-Bowan et al., 2020 | 1 | 0 | N/A | 2 | 2 | 0 | 1 | 6 | Moderate |
| Bediako-Bowan et al., 2020 (2) | 1 | 0 | N/A | 2 | 2 | 0 | 1 | 6 | Moderate |
| Behari et al., 2015 | 0 | 0 | N/A | 0 | 2 | 2 | 0 | 4 | Poor |
| Belay et al., 2022 | 1 | 1 | N/A | 2 | 2 | 0 | 1 | 7 | Good |
| Birhanu et al, 2022 | 1 | 1 | N/A | 2 | 2 | 0 | 1 | 7 | Good |
| Bizuayehu et al., 2022 | 1 | 0 | N/A | 1 | 0 | 2 | 1 | 5 | Moderate |
| Bizuayew et al., 2021 | 1 | 1 | N/A | 2 | 2 | 0 | 1 | 7 | Good |
| Bunduki et al, 2021 | 1 | 0 | N/A | 2 | 2 | 0 | 1 | 6 | Moderate |
| Chernet et al., 2020 | 1 | 0 | N/A | 2 | 2 | 2 | 1 | 8 | Good |
| Dawit et al., 2021 | 1 | 0 | N/A | 2 | 2 | 2 | 1 | 8 | Good |
| Dayyab et al., 2018 | 1 | 0 | N/A | 0 | 0 | 2 | 1 | 4 | Poor |
| De Nardo et al., 2016 | 1 | 0 | N/A | 2 | 2 | 0 | 1 | 6 | Moderate |
| Degbey et al., 2021 | 1 | 0 | N/A | 2 | 2 | 0 | 1 | 6 | Moderate |
| Di Gennaro et al., 2020 | 1 | 0 | N/A | 2 | 2 | 0 | 1 | 6 | Moderate |
| Dramowski et al., 2015 | 1 | 0 | N/A | 0 | 0 | 2 | 1 | 4 | Poor |
| Dramowski et al., 2016 | 1 | 0 | N/A | 0 | 0 | 2 | 1 | 4 | Poor |
| Endalafer et al., 2011 | 1 | 1 | N/A | 2 | 2 | 2 | 1 | 9 | Good |
| Fisha et al., 2019 | 1 | 1 | N/A | 2 | 2 | 0 | 1 | 7 | Good |
| Flouchi et al., 2022 | 1 | 0 | N/A | 2 | 2 | 0 | 1 | 6 | Moderate |
| Gadallah et al., 2014 | 1 | 0 | N/A | 2 | 2 | 2 | 1 | 8 | Good |
| Galal et al., 2016 | 1 | 0 | N/A | 2 | 2 | 0 | 1 | 6 | Moderate |
| Gelaw et al., 2017 | 1 | 1 | N/A | 2 | 2 | 0 | 1 | 7 | Good |
| Ghali et al., 2018 | 1 | 0 | N/A | 2 | 2 | 2 | 1 | 8 | Good |
| Gomaa et al., 2021 | 1 | 1 | N/A | 2 | 2 | 0 | 1 | 7 | Good |
| Hafez et al., 2012 | 1 | 0 | N/A | 2 | 2 | 2 | 1 | 8 | Good |
| Hajjej et al., 2014 | 1 | 0 | N/A | 2 | 2 | 2 | 1 | 8 | Good |
| Halawi et al., 2018 | 1 | 1 | N/A | 2 | 2 | 0 | 1 | 7 | Good |
| Hassan et al., 2020 | 1 | 0 | N/A | 2 | 2 | 2 | 1 | 8 | Good |
| Iwuafor et al., 2016 | 0 | 0 | N/A | 2 | 2 | 2 | 0 | 6 | Moderate |
| Jamoussi et al., 2018 | 1 | 0 | N/A | 2 | 2 | 0 | 1 | 6 | Moderate |
| Kakupa et al., 2016 | 1 | 0 | N/A | 0 | 0 | 2 | 0 | 3 | Poor |
| Kallel et al., 2010 | 1 | 0 | N/A | 1 | 2 | 0 | 1 | 5 | Moderate |
| Kefale et al., 2020 | 1 | 1 | N/A | 2 | 2 | 0 | 1 | 7 | Good |
| Ketata et al., 2021 | 1 | 0 | N/A | 2 | 2 | 2 | 1 | 8 | Good |
| Ketema et al., 2020 | 1 | 1 | N/A | 2 | 2 | 0 | 1 | 7 | Good |
| Kibwana et al., 2022 | 1 | 1 | N/A | 2 | 2 | 0 | 1 | 7 | Good |
| Kisibo et al., 2017 | 1 | 0 | N/A | 2 | 2 | 0 | 1 | 6 | Moderate |
| Labi et al., 2019 | 1 | 1 | N/A | 2 | 2 | 2 | 1 | 9 | Good |
| Lakoh et al., 2022 | 1 | 1 | N/A | 2 | 2 | 2 | 1 | 9 | Good |
| Lakoh et al., 2022 (2) | 1 | 1 | N/A | 2 | 2 | 2 | 1 | 9 | Good |
| Laloto et al., 2017 | 1 | 0 | N/A | 2 | 2 | 0 | 1 | 6 | Moderate |
| Lijaemiro et al., 2020 | 1 | 1 | N/A | 2 | 2 | 0 | 1 | 7 | Good |
| Lubega et al., 2017 | 1 | 1 | N/A | 1 | 2 | 2 | 1 | 8 | Good |
| Lukuke et al., 2017 | 1 | 0 | N/A | 0 | 0 | 2 | 1 | 4 | Poor |
| Mamo et al., 2017 | 1 | 1 | N/A | 2 | 2 | 0 | 1 | 7 | Good |
| Maoulainine et al., 2014 | 1 | 0 | N/A | 2 | 2 | 2 | 1 | 8 | Good |
| Mawalla et al., 2011 | 1 | 1 | N/A | 2 | 2 | 0 | 1 | 7 | Good |
| Melaku et al., 2012 | 1 | 0 | N/A | 1 | 2 | 2 | 1 | 7 | Good |
| Mezemir et al., 2020 | 1 | 1 | N/A | 2 | 2 | 0 | 1 | 7 | Good |
| Misha et al., 2021 | 1 | 1 | N/A | 1 | 2 | 2 | 1 | 8 | Good |
| Misha et al., 2021 (2) | 1 | 1 | N/A | 2 | 2 | 0 | 1 | 7 | Good |
| Mohamed et al., 2022 | 1 | 1 | N/A | 1 | 2 | 0 | 1 | 6 | Moderate |
| Molla et al., 2019 | 1 | 0 | N/A | 2 | 2 | 0 | 1 | 6 | Moderate |
| Mpogoro et al., 2014 | 1 | 1 | N/A | 2 | 2 | 0 | 1 | 7 | Good |
| Mukagendaneza et al., 2019 | 1 | 0 | N/A | 2 | 2 | 2 | 1 | 8 | Good |
| Nair et al., 2018 | 1 | 0 | N/A | 2 | 2 | 0 | 1 | 6 | Moderate |
| Nanyunja et al., 2022 | 1 | 0 | N/A | 1 | 2 | 2 | 1 | 7 | Good |
| Nkurunziza et al., 2019 | 1 | 0 | N/A | 2 | 2 | 0 | 1 | 6 | Moderate |
| Nouetchognou et al., 2016 | 1 | 0 | N/A | 0 | 0 | 2 | 1 | 4 | Poor |
| Nwanko et al. 2016 | 1 | 0 | N/A | 0 | 2 | 2 | 1 | 6 | Moderate |
| Olowo-okere et al., 2018 | 1 | 1 | N/A | 2 | 2 | 0 | 1 | 7 | Good |
| Ouedraogo et al., 2020 | 1 | 0 | N/A | 0 | 2 | 0 | 1 | 4 | Poor |
| Oumer et al., 2021 | 1 | 0 | N/A | 2 | 2 | 2 | 1 | 8 | Good |
| Raouf et al., 2020 | 1 | 0 | N/A | 2 | 2 | 2 | 1 | 8 | Good |
| Sahiledengle et al., 2020 | 1 | 0 | N/A | 2 | 2 | 0 | 1 | 6 | Moderate |
| Saied et al., 2011 | 1 | 0 | N/A | 0 | 0 | 2 | 1 | 4 | Poor |
| Salem et al., 2011 | 1 | 0 | N/A | 2 | 2 | 0 | 1 | 6 | Moderate |
| Sattar et al., 2018 | 0 | 0 | N/A | 1 | 2 | 2 | 1 | 6 | Moderate |
| Scherbaum et al., 2014 | 1 | 0 | N/A | 0 | 0 | 2 | 1 | 4 | Poor |
| See et al., 2013 | 1 | 0 | N/A | 0 | 0 | 2 | 1 | 4 | Poor |
| Shakir et al., 2021 | 1 | 1 | N/A | 2 | 2 | 0 | 1 | 7 | Good |
| Shimi et al., 2015 | 1 | 0 | N/A | 0 | 0 | 2 | 1 | 4 | Poor |
| Titus et al., 2021 | 1 | 1 | N/A | 2 | 2 | 0 | 1 | 7 | Good |
| Togo et al., 2010 | 1 | 1 | N/A | 0 | 0 | 2 | 1 | 5 | Moderate |
| Velin et al., 2021 | 1 | 0 | N/A | 0 | 0 | 2 | 1 | 4 | Poor |
| Victor et al., 2013 | 1 | 0 | N/A | 2 | 2 | 2 | 1 | 8 | Good |
| Weldu et al., 2018 | 1 | 1 | N/A | 2 | 2 | 0 | 1 | 7 | Good |
| Wendmagegn et al., 2018 | 1 | 1 | N/A | 2 | 2 | 0 | 1 | 7 | Good |
| Wodajo et al., 2017 | 1 | 1 | N/A | 2 | 2 | 0 | 1 | 7 | Good |
| Yallew et al., 2016 | 1 | 0 | N/A | 2 | 2 | 0 | 1 | 6 | Moderate |
| Yaouba et al., 2016 | 0 | 0 | N/A | 2 | 2 | 0 | 1 | 5 | Moderate |

# Table S4: CASP risk bias assessment

| **Authors** | **Domain 1** | **Domain 2** | **Domain 3** | **Domain 4** |
| --- | --- | --- | --- | --- |
| Abdel-Wahab et al., 2013 | partially | yes | partially | no |
| Abosse et al., 2021 | yes | yes | no | yes |
| Abubakar, 2020 | yes | yes | yes | partially |
| Ahoyo et al., 2014 | partially | yes | yes | partially |
| Akoko et al., 2012 | yes | partially | yes | partially |
| Alemye et al., 2021 | yes | partially | yes | partially |
| Atif et al., 2015 | yes | yes | yes | partially |
| Awoke et al., 2019 | yes | yes | no | partially |
| Ayala et al., 2021 | yes | yes | no | partially |
| Ayed et al., 2019 | yes | yes | yes | partially |
| Azeze et al., 2019 | yes | partially | no | partially |
| Bediako-Bowan et al., 2020 | yes | no | yes | partially |
| Bediako-Bowan et al., 2020 (2) | yes | partially | yes | partially |
| Behari et al., 2015 | partially | yes | no | no |
| Belay et al., 2022 | partially | yes | no | partially |
| Birhanu et al, 2022 | yes | partially | no | partially |
| Bizuayehu et al., 2022 | yes | yes | no | partially |
| Bizuayew et al., 2021 | yes | partially | yes | partially |
| Bunduki et al, 2021 | yes | yes | yes | partially |
| Chernet et al., 2020 | yes | yes | partially | yes |
| Dawit et al., 2021 | yes | partially | no | yes |
| Dayyab et al., 2018 | yes | yes | yes | partially |
| De Nardo et al., 2016 | yes | partially | yes | partially |
| Degbey et al., 2021 | yes | no | no | partially |
| Di Gennaro et al., 2020 | yes | partially | yes | partially |
| Dramowski et al., 2015 | partially | partially | yes | no |
| Dramowski et al., 2016 | yes | yes | yes | partially |
| Endalafer et al., 2011 | yes | yes | yes | partially |
| Fisha et al., 2019 | yes | partially | no | partially |
| Flouchi et al., 2022 | yes | yes | yes | partially |
| Gadallah et al., 2014 | partially | yes | yes | partially |
| Galal et al., 2016 | yes | yes | no | partially |
| Gelaw et al., 2017 | yes | partially | no | partially |
| Ghali et al., 2018 | yes | yes | yes | partially |
| Gomaa et al., 2021 | yes | partially | no | partially |
| Hafez et al., 2012 | yes | yes | yes | partially |
| Hajjej et al., 2014 | yes | yes | partially | yes |
| Halawi et al., 2018 | yes | yes | yes | partially |
| Hassan et al., 2020 | partially | partially | no | partially |
| Iwuafor et al., 2016 | yes | partially | no | yes |
| Jamoussi et al., 2018 | yes | partially | no | partially |
| Kakupa et al., 2016 | yes | no | partially | no |
| Kallel et al., 2010 | yes | yes | yes | no |
| Kefale et al., 2020 | yes | no | no | partially |
| Ketata et al., 2021 | yes | partially | yes | partially |
| Ketema et al., 2020 | partially | partially | yes | partially |
| Kibwana et al., 2022 | yes | partially | no | yes |
| Kisibo et al., 2017 | yes | partially | no | partially |
| Labi et al., 2019 | no | partially | yes | yes |
| Lakoh et al., 2022 | yes | partially | yes | yes |
| Lakoh et al., 2022 (2) | yes | partially | yes | yes |
| Laloto et al., 2017 | yes | yes | yes | partially |
| Lijaemiro et al., 2020 | yes | yes | no | partially |
| Lubega et al., 2017 | yes | no | no | yes |
| Lukuke et al., 2017 | yes | partially | partially | yes |
| Mamo et al., 2017 | yes | yes | yes | partially |
| Maoulainine et al., 2014 | yes | yes | yes | partially |
| Mawalla et al., 2011 | yes | partially | yes | partially |
| Melaku et al., 2012 | yes | partially | yes | partially |
| Mezemir et al., 2020 | yes | yes | yes | partially |
| Misha et al., 2021 | yes | partially | no | partially |
| Misha et al., 2021 (2) | yes | partially | yes | partially |
| Mohamed et al., 2022 | yes | yes | no | partially |
| Molla et al., 2019 | yes | yes | yes | partially |
| Mpogoro et al., 2014 | yes | yes | yes | partially |
| Mukagendaneza et al., 2019 | yes | yes | yes | yes |
| Nair et al., 2018 | yes | partially | yes | partially |
| Nanyunja et al., 2022 | yes | no | no | yes |
| Nkurunziza et al., 2019 | yes | partially | no | partially |
| Nouetchognou et al., 2016 | yes | no | no | partially |
| Nwanko et al. 2016 | no | no | no | no |
| Olowo-okere et al., 2018 | yes | partially | yes | partially |
| Ouedraogo et al., 2020 | yes | partially | yes | no |
| Oumer et al., 2021 | yes | yes | yes | yes |
| Raouf et al., 2020 | yes | partially | yes | partially |
| Sahiledengle et al., 2020 | partially | partially | yes | partially |
| Saied et al., 2011 | no | partially | yes | partially |
| Salem et al., 2011 | partially | no | yes | partially |
| Sattar et al., 2018 | yes | no | no | partially |
| Scherbaum et al., 2014 | yes | partially | partially | partially |
| See et al., 2013 | yes | partially | partially | no |
| Shakir et al., 2021 | yes | yes | no | partially |
| Shimi et al., 2015 | yes | partially | yes | partially |
| Titus et al., 2021 | yes | partially | no | partially |
| Togo et al., 2010 | no | partially | yes | partially |
| Velin et al., 2021 | partially | partially | no | partially |
| Victor et al., 2013 | yes | partially | yes | yes |
| Weldu et al., 2018 | yes | yes | yes | partially |
| Wendmagegn et al., 2018 | yes | no | no | partially |
| Wodajo et al., 2017 | yes | no | yes | partially |
| Yallew et al., 2016 | yes | partially | yes | partially |
| Yaouba et al., 2016 | yes | partially | yes | partially |

# Figure S1: Forest plot of good quality studies pooled by HCAI types

**A. Pneumonia**


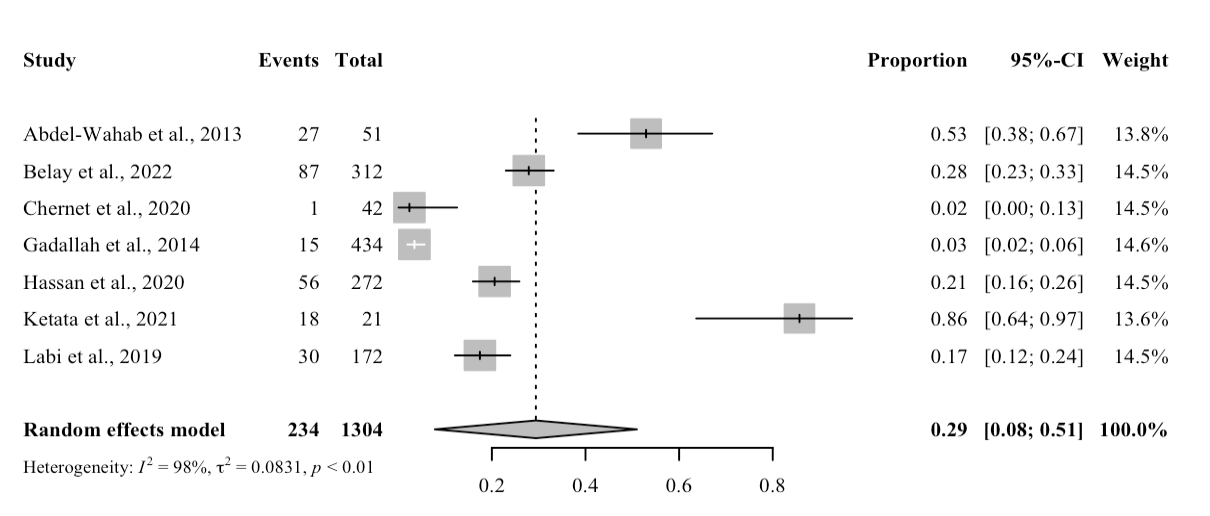
 **B. Bloodstream infection**


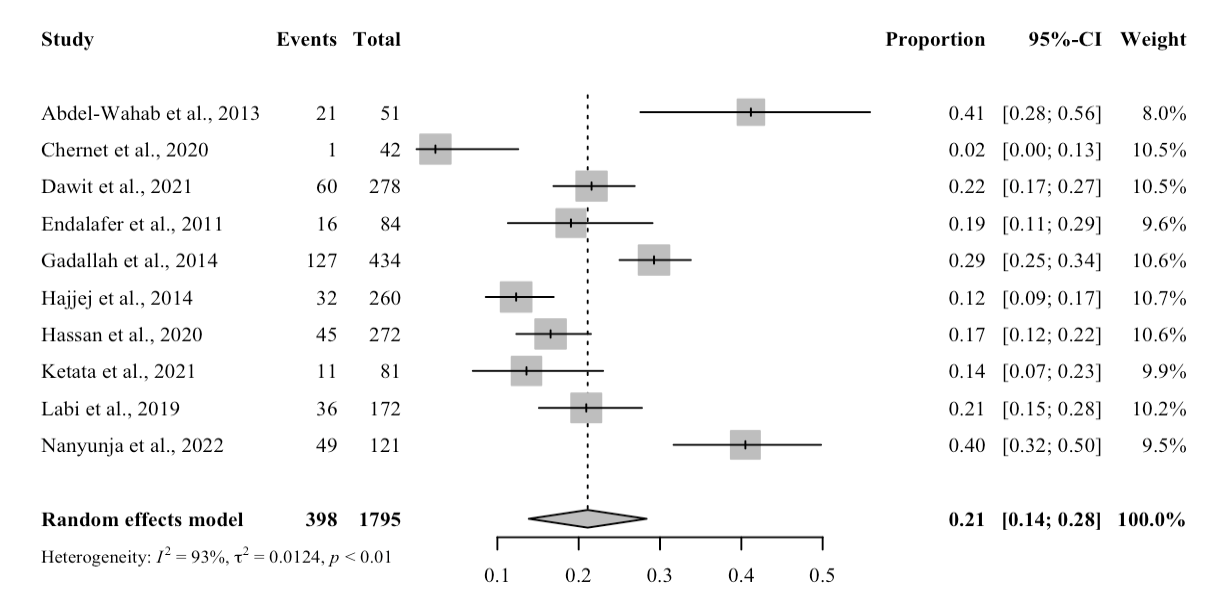


**C. Urinary tract infection**


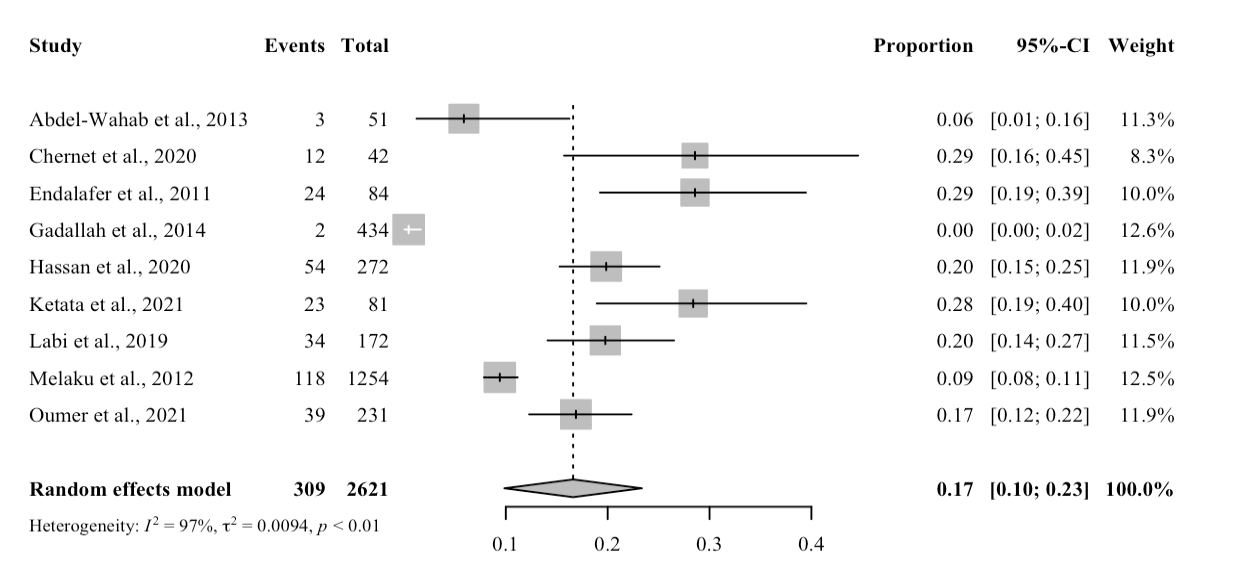


**D. Surgical site infection**


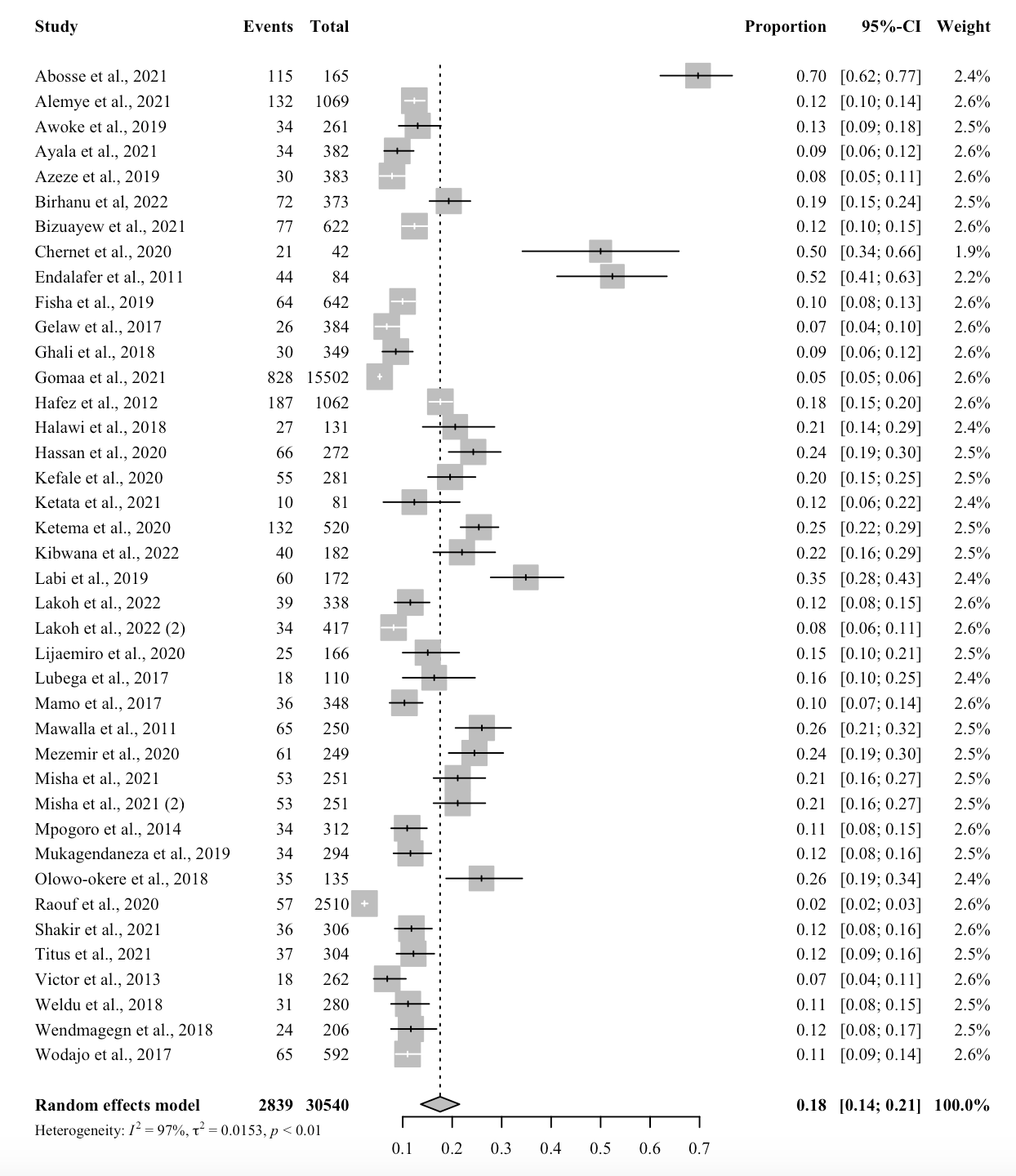


# Table S5: Significant risk factors reported in included studies

| **Authors** | **Analysis** | **Significant risk factors** | **OR** | **95% CI** |
| --- | --- | --- | --- | --- |
| Mawalla et al., 2011 | mv | Presence of pre-morbid illness | 6.1 | 1.3-28.9 |
|  |  | Use of drain | 3.5 | 1.4-19.3 |
|  |  | Use of iodine alone in skin preparation | 17.6 | 6.5-48.1 |
|  |  | Duration of operation >2 hours | 3.2 | 1.1-9.3 |
|  |  | Cigarette smoking | 9.6 | 2.4-38.3 |
| Salem et al., 2011 | mv | Diabetes | 2 | 1.03-3.02 |
|  |  | Length of stay | 4.5 | 2.47-8.24 |
|  |  | Immunosuppression | 3.3 | 1.17-9.27 |
|  |  | CVC | 2.53 | 1.50-4.28 |
|  |  | PVC | 10.2 | 4.25-24.5 |
| Endafar et al., 2011 | mv | Age >65y | 1.05 | 0.85-1.40 |
|  |  | Antibiotic prophylaxis | 0.96 | 0.72-1.17 |
|  |  | Surgical procedures | 3.96 | 2.96-5.25 |
| Melaku et al., 2012 | uv | Catheter insertion | 18.9 | 2.54-15.7 |
|  |  | Antibiotic prophylaxis | 1.2 | 1.33-2.43 |
|  |  | Underlying diseases | 4.3 | 2.73-6.69 |
| Hafez et al., 2012 | mv | Underwent thoracic surgery | 4.7 | 2.2-11.1 |
|  |  | Age>45y | 2.32 | 1.35-4.01 |
|  |  | Increased hospital stay before surgery | 1.03 | 1.01-1.05 |
|  |  | Increased hospital stay after surgery | 1.07 | 1.04-1.09 |
|  |  | Antibiotic <24h before surgery | 2.54 | 1.63-3.94 |
|  |  | Dirty wound | 4.09 | 1.60-10.43 |
| Akoko et al., 2012 | uv | Dirty wound | 36.9 | 6.15-221.35 |
|  |  | Emergency procedure | 3.08 | 0.98-9.69 |
| Mpogoro et al., 2014 | mv | Hypertensive disorders of pregnancy | 2.9 | 1.4-6.4 |
|  |  | Contaminated wound | 2.5 | 1.2-5.1 |
|  |  | Multiple vaginal examinations | 2.6 | 1.3-5.3 |
|  |  | Operation done by intern doctor | 4.2 | 1.8-9.5 |
|  |  | Severe anaemia (Hb<7g/dl) | 3.8 | 1.2-12.4 |
|  |  | Duration of procedure >60 minutes | 2.3 | 1.1-4.8 |
| Gadallah et al., 2014 | uv | Gestational age <38 weeks | 1.86 | 1.24-2.79 |
|  |  | Birth weigh <1500 g | 1.64 | 1.22-2.22 |
|  |  | CVC | 1.9 | 1.42-2.57 |
|  |  | Mechanical ventilation | 1.99 | 1.44-2.74 |
| Hajjej et al., 2014 | mv | Diabetes | 2.43 | 1.09-5.7 |
|  |  | Duration of catheterization | 1.95 | 1.21-2.13 |
|  |  | Sepsis at catheter insertion | 3.8 | 1.91-7.87 |
|  |  | One or more antibiotics before insertion | 4.46 | 2.08-10.1 |
| Atif et al., 2015 | mv | NNIS risk index | 2.91 | 1.56-4.30 |
|  |  | Age | 1.3 | 1.04-1.68 |
| Iwuafor et al., 2016 | uv | Use of antibiotics | 2.98 | 0.71-6.50 |
|  |  | History of surgery in the past 30d | 3.15 | 1.08-13.52 |
|  |  | Urethral catheterization | 5.38 | 0.11-29.86 |
|  |  | Endotracheal intubation | 5.78 | 0.58-13.17 |
| Yallew et al., 2016 | mv | History of surgery in the past 30d | 2.86 | 1.72-4.78 |
|  |  | Age 1-14y | 0.25 | 0.09-0.71 |
| Galal et al., 2016 | mv | Multiple organ system failure | 4.62 | 2.90-7.35 |
|  |  | Previous antibiotics >48h | 2.26 | 1.41-3.62 |
|  |  | Re-intubation | 1.79 | 1.08-2.99 |
|  |  | Coma | 1.73 | 1.05-2.86 |
|  |  | age in months | 0.99 | 0.98-0.998 |
| Yaouba et al., 2016 | mv | ASA >2 | 8.1 | 2.2-30.3 |
|  |  | Hair removal inside the operating room | 5.8 | 1.5-23.2 |
|  |  | Preoperative showering | 0.25 | 0.05-1.30 |
| De Nardo et al., 2016 | uv | Business profession | 0.6 | 0.38-0.94 |
|  |  | Emergency procedure | 0.79 | 0.42-1.50 |
|  |  | Desinfection by povidone/iodine | 1.23 | 0.78-1.97 |
| Lubega et al., 2017 | mv | Hemoglobin levels | 2.4 | 1.12-5.34 |
|  |  | Serum albumin | 4.3 | 1.02-17.84 |
|  |  | Suture materiel | 30.7 | 2.7-351.31 |
|  |  | Wound classification | 2.59 | 1,27-5.27 |
| Laloto et al., 2017 | mv | Age >40y | 7.72 | 1.46-40.81 |
|  |  | Preoperative stay >7d | 22.4 | 4.54-110.78 |
|  |  | Duration of procedure >60 minutes | 8.01 | 1.56-41.01 |
|  |  | Time of 1st dose AMP before skin incision | 11.1 | 1.63-75.64 |
| Wodajo et al., 2017 | mv | Duration of labour >24h | 6.78 | 2.54-18 |
|  |  | Membrane rupture >12h | 5.83 | 2.14-15.89 |
|  |  | Multiple vaginal examinations | 8.59 | 1.74-42.23 |
|  |  | Duration of procedure >60 minutes | 12.32 | 5.46-27.77 |
|  |  | Wound classification III | 9.61 | 1.84-50.06 |
|  |  | Operation done by resident | 8.31 | 1.79-38.52 |
|  |  | Postoperative hemoglobin <11mg/dl | 2.62 | 1.21-5.69 |
| Gelaw et al., 2017 | mv | Duration of labour >24h | 3.48 | 1.25-9.68 |
|  |  | Membrane rupture >12h | 3.68 | 1.13-11.96 |
|  |  | Midline incision | 5.73 | 2.05-16.00 |
| Mamo et al., 2017 | mv | Age <19y | 3.5 | 1.17-10.01 |
|  |  | Preterm gestation age | 4.23 | 1.26-14.24 |
|  |  | Duration of labour >24h | 2.22 | 1.04-4.67 |
|  |  | Membrane rupture >12h | 5.99 | 2.75-13.02 |
|  |  | Chorioamniotis | 9.74 | 3.08-30.85 |
|  |  | Mdline incision | 4.95 | 1.71-13.32 |
|  |  | Preoperation hematocrit | 6.4 | 1.02-40.14 |
|  |  | perioperativr blood transfusion | 6.75 | 2.47-18.49 |
|  |  | Abdominal hysterectomy | 7.9 | 1.70-36.96 |
|  |  | Diabetes mellitus | 3.7 | 1.11-12.52 |
| Kisibo et al., 2017 | mv | Duration of operation >2 hours | 1.4 | 1.14-6.69 |
|  |  | Antibiotic prophylaxis not used | 3.4 | 1.6-7.78 |
|  |  | Time of stay before surgery >7d | 3.3 | 2.24-3.34 |
| Olowo-okere et al., 2018 | mv | Postoperative hospital stays | 1.07 | 1.01-1.13 |
| Ghali et al., 2018 | mv | Preoperative length of stay | 1.2 | 1.06-1.36 |
|  |  | NNIS risk index 1 | 45.45 | 14.70-142-85 |
|  |  | NNIS risk index 2 | 10.1 | 3.28-31.25 |
| Weldu et al., 2018 | mv | Postoperative hospital stays 8-14d | 7.97 | 1.70-37.38 |
|  |  | History of alcohol use | 0.04 | 0.004-0.43 |
|  |  | Use of local anesthesia | 8 | 1.01-63.40 |
|  |  | Dirty wound | 17 | 1.249-232.362 |
| Wendmagegn et al., 2018 | mv | Rural residence | 5.67 | 1.568-20.483 |
|  |  | Grand multi parity | 9.55 | 1.519-60.075 |
|  |  | Membrane rupture >12h | 8.82 | 2.171-35.816 |
|  |  | Duration of labour >24h | 6.06 | 1.676-21.949 |
|  |  | Chorioamniotis | 16.17 | 2.850-91.819 |
|  |  | HIV | 6.98 | 1.382-35.269 |
| Halawi et al., 2018 | mv | History of alcohol use | 7.7 | 2.56-23.13 |
|  |  | History of surgery in the past 30d | 3.22 | 1.14-9.13 |
| Nair et al., 2018 | uv | History of surgery in the past 30d | 4.12 | 1.79-9.49 |
| Mukagendaneza et al., 2019 | uv | ASA >2 | 3.9 | 1.9-8.1 |
|  |  | Contaminated wound and dirty wound | 8.5 | 2-35.9 |
|  |  | Operation done by resident | 2.2 | 1.1-4.6 |
|  |  | Duration of operation >2 hours | 2.2 | 1.1-4.6 |
|  |  | Transfusion | 6.8 | 2.9-16.3 |
|  |  | Postoperative hospital stays >14d | 42.3 | 16.4-108.9 |
| Labi et al., 2019 | mv | Duration of hospital stay before HAI onset | 2.78 | 2.08-10.18 |
|  |  | Surgery since admission | 1.79 | 1.35-2.98 |
|  |  | Presence of any invasive device | 5.03 | 4.77-13.35 |
|  |  | Urinary catheter in place during hospitalisation | 1.76 | 1.13-3.30 |
|  |  | PVC | 1.73 | 1.22-2.57 |
|  |  | Patient/consultant speciality (surgery) | 1.81 | 1.26-2.59 |
|  |  | Admission to a primary care hospital | 0.37 | 0.33-0.71 |
| Molla et al., 2019 | mv | Pregnancy induced hypertension | 4.75 | 1.62-13.92 |
|  |  | Chorioamniotis | 4.37 | 1.53-12.50 |
|  |  | Midline incision | 5.19 | 1.87-14.37 |
|  |  | Postoperative hemoglobin <11mg/dl | 5.28 | 1.97-14.18 |
| Ayed et al., 2019 | mv | Immunosuppression | 2.8 | 1.6-5 |
|  |  | Malnutrition | 2.2 | 1.1-4.3 |
|  |  | Diabetes | 2.2 | 1.2-3.9 |
|  |  | Endotracheal intubation | 17 | 3.2-90 |
|  |  | Transfer to another department | 9 | 1.8-37 |
|  |  | Parenteral feeding | 7.2 | 1.4-35 |
|  |  | Contaminated wound and dirty wound | 6.3 | 2.0-20 |
|  |  | PVC | 4.7 | 1.56-14 |
|  |  | Tobacco use | 6.3 | 1.7-22.3 |
| Fisha et al., 2019 | mv | Contaminated wound and dirty wound | 5.35 | 2.84-10.06 |
|  |  | Length of stay >6d | 1.96 | 1.03-3.71 |
|  |  | Duration of procedure >60 minutes | 5.36 | 2.09-13.75 |
|  |  | Operation done by resident and GP | 3.99 | 2.01-7.91 |
| Nkurunziza et al., 2019 | mv | Bodyweight >75kgs | 5.98 | 1.56-22.96 |
|  |  | Skin preparation with one antiseptic | 4.42 | 1.05-18.57 |
| Azeze et al., 2019 | mv | Duration of operation >30 min | 4.9 | 1.8-13.1 |
|  |  | Membrane rupture >12h | 13.9 | 2.99-64.8 |
|  |  | Midline incision | 4.77 | 1.74-13.06 |
|  |  | Suture technique interrupted | 6.29 | 2.07-19.11 |
| Awoke et al., 2019 | mv | Age >40y | 6.45 | 1.56-26.67 |
|  |  | Illiterate | 4.25 | 1.52-11.84 |
|  |  | History of previous hospitalisation | 4.5 | 1.44-14.08 |
|  |  | Public ward | 0.24 | 0.07-0.79 |
|  |  | Preoperative stay >7d | 3.88 | 1.46-10.29 |
| Dramowski et al., 2016 | mv | Admission in ICU | 7.6 | 3.3-17.6 |
|  |  | Transfusion | 8.1 | 3.9-16.6 |
|  |  | Stay in an isolation room | 7.6 | 2.9-19.6 |
| Abubakar, 2020 | uv | Age <1month | 4.69 | 1.298-16.927 |
|  |  | Intubation | 3.97 | 1.698-9.261 |
|  |  | Neonatal speciality | 41.54 | 4.980-346.5 |
|  |  | Pediatric surgery | 13.09 | 1.532-111.874 |
| Sahiledengle et al., 2020 | mv | Length of stay >6d | 2.58 | 1.52-4.38 |
|  |  | Malnutrition | 2.83 | 1.61-4.97 |
| Hassan et al., 2020 | mv | Multiple devices | 88.1 | 56.9-136.5 |
|  |  | CVC | 34 | 19.7-58.4 |
|  |  | Urethral catheterization | 28.9 | 19.5-43 |
|  |  | Length of stay >20d | 3.1 | 2.2-4.4 |
| Bediako-Bowan et al., 2020 | mv | ASA >2 | 1.5 | 1.0-2.1 |
|  |  | Contaminated wound and dirty wound | 2.2 | 1.4-3.7 |
| Chernet et al., 2020 | mv | Diabetes | 0.207 | 0.06-0.79 |
|  |  | Renal disease | 0.194 | 0.05-0.73 |
| Kefale et al., 2020 | mv | Comorbidity | 9.18 | 5.17-17.9 |
|  |  | Antibiotic prophylaxis not used | 6.63 | 1.89-19.3 |
|  |  | Prophylaxy 1 to 2h before incision | 8.2 | 4.34-18.1 |
|  |  | Duration of SAP >2d | 7.2 | 1.23-28.17 |
| Ketema et al., 2020 | mv | Not able to read and write | 1.3 | 1.19-2.11 |
|  |  | No antenatal care | 2.16 | 1.05-4.53 |
|  |  | Previous history of CS | 1.21 | 1.11-2.31 |
|  |  | HIV | 1.39 | 1.21-2.57 |
|  |  | Emergency procedure | 1.13 | 1.11-2.43 |
|  |  | Midline incision | 2.6 | 1.05-6.44 |
|  |  | Membrane rupture >12h | 1.5 | 1.31-1.64 |
|  |  | Multiple vaginal examinations | 1.88 | 1.71-3.20 |
| Raouf et al., 2020 | uv | Cigarette smoking | 2.58 | 1.49-4.47 |
|  |  | Rural residence | 5.03 | 2.37-10.66 |
|  |  | Diabetes | 5.29 | 3.09-9.05 |
|  |  | Cardiovascular diseases | 2.53 | 1.06-6.02 |
|  |  | Organ failure | 5.8 | 2.20-15.29 |
|  |  | Previous admission | 2.23 | 1.32-3.77 |
|  |  | Associated infection | 9.36 | 5.31-16.52 |
|  |  | Not Showering | 2.79 | 1.64-4.76 |
|  |  | ASA 5 | 6.86 | 3.11-15.16 |
|  |  | Postoperative hospital stays >30d | 26.27 | 10.1-68.7 |
| Mezemir et al., 2020 | mv | Ctertiary education | 0.1 | 0.03-0.913 |
|  |  | Preoperative stay >4h | 6 | 1.5-27.90 |
|  |  | Postoperative hospital stays 7-14d | 4.3 | 1.11-16.10 |
|  |  | Postoperative hospital stays >14d | 5 | 2.04-101.12 |
|  |  | ASA 1 | 0.3 | 0.07-1.26 |
|  |  | Contaminated wound and dirty wound | 6 | 5.39-35.94 |
| Lijaemiro et al., 2020 | mv | Age>30y | 1.5 | 1.170-1.933 |
|  |  | Multiple vaginal examinations | 13.08 | 1.018-168.002 |
| Abosse et al., 2021 | mv | Contaminated wound and dirty wound | 36.4 | 7.115-185.281 |
|  |  | Duration of hospital stay >14d | 4.2 | 1.446-12.009 |
| Misha et al., 2021 | mv | ASA >2 | 2.26 | 1.03-4.93 |
|  |  | Postoperative antibiotic prescription | 3.2 | 1.71-6.01 |
|  |  | Contaminated wound and dirty wound | 7.9 | 4.3-14.60 |
|  |  | Emergency procedure | 2.8 | 1.16-6.80 |
|  |  | Duration of operation >2 hours | 4 | 2.17-7.50 |
|  |  | Comorbidity | 2.52 | 1.28-4.94 |
| Ketata et al., 2021 | mv | Diabetes | 3.5 | 1.2-9.9 |
|  |  | McCabe Ultimately fatal disease | 4 | 1.2-13.7 |
|  |  | McCabe Rapidly fatal disease | 2.8 | 1.1-7.7 |
|  |  | Admission in ICU | 11.1 | 2.1-58.2 |
|  |  | Length of stay >6d | 4.1 | 1.1-17.3 |
|  |  | Previous admission | 4.2 | 1.4-12.9 |
|  |  | Antibiotic prophylaxis not used | 0.1 | 0.02-0.7 |
|  |  | PVC | 6.7 | 1.1-40 |
| Oumer et al., 2021 | mv | Urethral catheterization | 3.8 | 1.1-13.61 |
|  |  | Catheterization >7d | 3.08 | 1.2-7.4 |
|  |  | Diabetes | 5.1 | 1.4-19 |
| Degbey et al., 2021 | mv | Transfer by another center | 2.74 | 1.08-6.95 |
|  |  | Duration of postoperative stay >14d | 3.55 | 1.07-11.75 |
|  |  | Duration of postoperative stay >21d | 4.7 | 1.22-18.11 |
|  |  | Duration of postoperative stay >5 | 8.75 | 2.83-26.98 |
| Bizuayew et al., 2021 | mv | Rural residence | 2.3 | 1.29-4.09 |
|  |  | Duration of labour >24h | 3.48 | 1.49-8.09 |
|  |  | Membrane rupture >12h | 4.61 | 2.34-9.09 |
|  |  | Hypertensive disorders of pregnancy | 3.14 | 1.29-7.59 |
|  |  | Preoperation hematocrit <30% | 3.22 | 1.25-8.31 |
| Dawit et al., 2021 | mv | Mechanical ventilation | 5.7 | 2.6-12.7 |
|  |  | Length of stay >7d | 9.3 | 4.3-20.4 |
|  |  | CVC | 1.1 | 0.3-3.7 |
| Titus et al., 2021 | mv | Contaminated wound and dirty wound | 6.54 | 1.78-24.04 |
|  |  | Operation time >4h | 8.35 | 1.31-53.18 |
|  |  | Gastrointestinal repair | 9.69 | 2.4-39.24 |
|  |  | HIV | 13.29 | 3.63-88.63 |
| Ayala et al., 2021 | mv | Age>35y | 5.03 | 1.69-14.95 |
|  |  | Hypertensive disorders of pregnancy | 5.63 | 1.88-16.79 |
|  |  | Duration of labour >24h | 4.12 | 1.01-32.19 |
|  |  | General anesthesia | 3.96 | 1.02-15.29 |
|  |  | Postoperative hemoglobin <11mg/dl | 4.51 | 1.84-11.07 |
| Shakir et al., 2021 | mv | Wound with drain | 24.54 | 10.053-58.898 |
|  |  | Diabetis | 7.457 | 2.89-19.22 |
|  |  | Age>60y | 4.139 | 1.278-13.40 |
|  |  | Duration of operation >2 hours | 0.159 | 0.040-0.630 |
|  |  | Alcohol drinker | 2.58 | 1.091-6.102 |
|  |  | Dirty wound | 9.026 | 3.503-23.255 |
| Gomaa et al., 2021 | mv | Membrane rupture >12h | 3.99 | 3.11-4.74 |
|  |  | Blood loss >1000ml | 2.21 | 1.62-3.09 |
|  |  | No antenatal care | 2.05 | 1.66-2.37 |
|  |  | Duration of labour >24h | 1.45 | 1.06-2.01 |
|  |  | Diabetes | 1.37 | 1.02-2.13 |
|  |  | Obesity | 1.34 | 0.95-1.84 |
|  |  | High parity | 1.27 | 1.03-1.88 |
|  |  | Hypertension | 1.19 | 0.92-2.11 |
|  |  | Gestational age <37 weeks | 1.12 | 0.94-1.66 |
| Alemye et al., 2021 | mv | General anesthesia | 2 | 1.10-2.90 |
|  |  | Membrane rupture >12h | 2.27 | 1.02-3.52 |
|  |  | Duration of hospital stay after operation >7d | 3.57 | 1.91-5.21 |
|  |  | Blood transfusion | 4.2 | 2.35-6.08 |
| Bunduki et al, 2021 | mv | Length of stay 8-14 days | 14.4 | 1.65-124.7 |
|  |  | Indwelling urinary catheter | 8.3 | 2.24-30.70 |
|  |  | History of surgery in the past 30d | 5.11 | 1.46-17.83 |
| Birhanu et al, 2022 | mv | Blood transfusion | 0.16 | 0.04-0.73 |
|  |  | Hemoglobin levels <7g/dl | 10.4 | 3.39-32.49 |
|  |  | Shock | 19.09 | 4.69-77.51 |
|  |  | Previous surgery | 11.53 | 3.73-35.61 |
|  |  | Length of stay 7-14 days | 5.51 | 1.52-19.91 |
|  |  | Hospitalisation >14 | 8.18 | 1.84-36.75 |
| Kibwana et al., 2022 | mv | Preoperative UTI | 9.73 | 3.93-24.09 |
|  |  | Contaminated wound and dirty wound | 24.997 | 2.58-242.42 |
| Lakoh et al., 2022 | mv | ASA >2 | 4.1 | 1.26-13.50 |
|  |  | Preoperative admission > 24h | 4.1 | 1.47-11.66 |
|  |  | General anesthesia | 6.8 | 1.40-33.08 |
|  |  | Number of surgeons >2 | 0.3 | 0.09-0.97 |
|  |  | Contaminated wound | 6.5 | 1.04-40.20 |
|  |  | Impaired glucose tolerance | 3.5 | 1.10-10.91 |
| Lakoh et al., 2022 (2) | mv | Contaminated wound and dirty wound | 6.82 | 1.66-28.1 |
| Mohamed et al., 2022 | mv | Length of stay >14d | 4.1 | 2.0-8.6 |
|  |  | Mechanical ventilation | 3.46 | 1.44-9.81 |
|  |  | Nasogastric tube feeding | 2.67 | 1.37-9.64 |
| Belay et al., 2022 | mv | Mechanical ventilation | 13.23 | 19.27-31.09 |
|  |  | Steroid use | 2.14 | 1.1-4.1 |
|  |  | Supine position | 8.1 | 1.66-39.6 |
|  |  | Blood transfusion | 2.78 | 1.13-6.86 |
|  |  | Low GCS | 2.5 | 1.27-5.1 |
| Flouchi et al., 2022 | uv | Wound with drain | 3.21 | 2.25-4.54 |
|  |  | Urethral catheterization | 0.52 | 0.37-0.72 |
| Bediako-Bowan et al., 2020 (2) | uv | Dirty wound | 3.15 | 0.94-10.62 |
|  |  | Duration of procedure >60 minutes | 2.48 | 1.19-5.52 |
| Di Gennaro et al., 2020 | mv | Single | 1.48 | 1.36-1.66 |
|  |  | Low BMI <18 | 1.42 | 1.18-1.72 |
|  |  | High BMI >25 | 1.85 | 1.02-2.68 |
|  |  | Referred from other health facilities | 2.35 | 2.18-2.59 |
|  |  | Gravida>4 | 0.64 | 0.59-0.83 |
|  |  | Unemployed | 1.74 | 1.24-2.21 |
|  |  | Low education | 2.19 | 1.71-2.33 |
|  |  | Membrane rupture >12h | 1.49 | 1.18-1.88 |
|  |  | Missing post-CS antibiotic use | 2.52 | 2.1-2.85 |
|  |  | Previous surgery | 1.27 | 1.1-1.52 |
| Nanyunja et al., 2022 | mv | Age>60y | 0.46 | 0.22-0.96 |
|  |  | Previous infection | 2.47 | 1.1-5.54 |
|  |  | Hemoglobin levels <11 | 5.44 | 1.32-22.48 |
| Jamoussi et al., 2018 | mv | Recent hospitalization | 4.4 | 1.24-13.89 |
|  |  | SAPS II >3 | 1.047 | 1.02-1.07 |

mv: multivariate analysis,

uv: univariate analysis

# Table S6: Pooled risk factors associated with HCAI in Africa

| **Risk factors** | **Authors** | **OR** | **95% CI Lim<** | **95% CI Lim>** |
| --- | --- | --- | --- | --- |
| Duration of procedure >60 minutes | Mawalla et al., 2011 | 3.2 | 1.1 | 9.3 |
|  | Laloto et al., 2017 | 8.01 | 1.56 | 41.01 |
|  | Wodajo et al., 2017 | 12.32 | 5.46 | 27.77 |
|  | Kisibo et al., 2017 | 1.4 | 1.14 | 6.69 |
|  | Mukagendaneza et al., 2019 | 2.2 | 1.1 | 4.6 |
|  | Fisha et al., 2019 | 5.36 | 2.09 | 13.75 |
|  | Misha et al., 2021 | 4 | 2.17 | 7.5 |
|  | Shakir et al., 2021 | 0.159 | 0.04 | 0.63 |
|  | Bediako-Bowan et al., 2020 | 2.48 | 1.19 | 5.52 |
|  | Mpogoro et al., 2014 | 2.3 | 1.1 | 4.8 |
|  | Titus et al., 2021 | 8.35 | 1.31 | 53.18 |
|  |  |  |  |  |
| Diabetes | Salem et al., 2011 | 2 | 1.03 | 3.02 |
|  | Hajjej et al., 2014 | 2.43 | 1.09 | 5.7 |
|  | Mamo et al., 2017 | 3.7 | 1.11 | 12.52 |
|  | Ayed et al., 2019 | 2.2 | 1.2 | 3.9 |
|  | Chernet et al., 2020 | 0.207 | 0.06 | 0.79 |
|  | Raouf et al., 2020 | 5.29 | 3.09 | 9.05 |
|  | Ketata et al., 2021 | 3.5 | 1.2 | 9.9 |
|  | Oumer et al., 2021 | 5.1 | 1.4 | 19 |
|  | Shakir et al., 2021 | 7.457 | 2.893 | 19.221 |
|  | Gomaa et al., 2021 | 1.37 | 1.02 | 2.13 |
|  |  |  |  |  |
| Contaminated and dirty wound | Hafez et al., 2012 | 4.09 | 1.6 | 10.43 |
|  | Akoko et al., 2012 | 36.9 | 6.15 | 221.35 |
|  | Mpogoro et al., 2014 | 2.5 | 1.2 | 5.1 |
|  | Lubega et al., 2017 | 2.59 | 1.27 | 5.27 |
|  | Wodajo et al., 2017 | 9.61 | 1.84 | 50.06 |
|  | Weldu et al., 2018 | 17 | 1.249 | 232.362 |
|  | Mukagendaneza et al., 2019 | 8.5 | 2 | 35.9 |
|  | Ayed et al., 2019 | 6.3 | 2 | 20 |
|  | Fisha et al., 2019 | 5.35 | 2.84 | 10.06 |
|  | Bediako-Bowan et al., 2020 | 2.2 | 1.4 | 3.7 |
|  | Mezemir et al., 2020 | 6 | 5.39 | 35.94 |
|  | Abosse et al., 2021 | 36.4 | 7.115 | 185.281 |
|  | Misha et al., 2021 | 7.9 | 4.3 | 14.6 |
|  | Titus et al., 2021 | 6.54 | 1.78 | 24.04 |
|  | Kibwana et al., 2022 | 24.997 | 2.58 | 242.42 |
|  | Lakoh et al., 2022 | 6.5 | 1.04 | 40.2 |
|  | Lakoh et al., 2022 (2) | 6.82 | 1.66 | 28.1 |
|  | Bediako-Bowan et al., 2020 (2) | 3.15 | 0.94 | 10.62 |
|  | Shakir et al., 2021 | 9.026 | 3.503 | 23.255 |
|  |  |  |  |  |
| Haemoglobin level <11g/dl | Mpogoro et al., 2014 | 3.8 | 1.2 | 12.4 |
|  | Wodajo et al., 2017 | 2.62 | 1.21 | 5.69 |
|  | Molla et al., 2019 | 5.28 | 1.97 | 14.18 |
|  | Bizuayew et al., 2021 | 3.22 | 1.25 | 8.31 |
|  | Ayala et al., 2021 | 4.51 | 1.84 | 11.07 |
|  | Birhanu et al., 2022 | 10.4 | 3.39 | 32.49 |
|  | Nanyunja et al., 2022 | 5.44 | 1.32 | 22.48 |
|  | Mamo et al., 2017 | 6.4 | 1.02 | 40.14 |
|  | Lubega et al., 2017 | 2.4 | 1.12 | 5.34 |
|  |  |  |  |  |
| Length of stay (pre or post-operative) >7 days | Salem et al., 2011 | 4.5 | 2.47 | 8.24 |
|  | Hafez et al., 2012 | 1.07 | 1.04 | 1.09 |
|  | Laloto et al., 2017 | 22.4 | 4.54 | 110.78 |
|  | Kisibo et al., 2017 | 3.3 | 2.24 | 3.34 |
|  | Olowo-okere et al., 2017 | 1.07 | 1.01 | 1.13 |
|  | Ghali et al., 2018 | 1.2 | 1.06 | 1.36 |
|  | Weldu et al., 2018 | 7.97 | 1.7 | 37.38 |
|  | Mukagendaneza et al., 2019 | 42.3 | 16.4 | 108.9 |
|  | Labi et al., 2019 | 2.78 | 2.08 | 10.18 |
|  | Fisha et al., 2019 | 1.96 | 1.03 | 3.71 |
|  | Awoke et al., 2019 | 3.88 | 1.46 | 10.29 |
|  | Sahikedengle et al., 2020 | 2.58 | 1.52 | 4.38 |
|  | Hassan et al., 2020 | 3.1 | 2.2 | 4.4 |
|  | Raouf et al., 2020 | 26.27 | 10.1 | 68.7 |
|  | Mezemir et al., 2020 | 6 | 1.5 | 27.9 |
|  | Abosse et al., 2021 | 4.2 | 1.446 | 12.009 |
|  | Ketata et al., 2021 | 4.1 | 1.1 | 17.3 |
|  | Degbey et al., 2021 | 8.75 | 22.83 | 26.98 |
|  | Dawit et al., 2021 | 9.3 | 4.3 | 20.4 |
|  | Alemye et al., 2021 | 3.57 | 1.91 | 5.21 |
|  | Bunduki et al., 2021 | 14.4 | 1.65 | 124.7 |
|  | Birhanu et al., 2021 | 8.18 | 1.84 | 36.75 |
|  | Mohamed et al., 2022 | 4.1 | 2 | 8.6 |
|  |  |  |  |  |
| History of surgery in the the past 30 days | Endafar et al., 2011 | 3.96 | 2.96 | 5.25 |
|  | Hafez et al., 2012 | 4.7 | 2.2 | 11.1 |
|  | Iwuafor et al., 2016 | 3.15 | 1.08 | 13.52 |
|  | Yallew et al., 2016 | 2.86 | 1.72 | 4.78 |
|  | Halawi et al., 2018 | 3.22 | 1.14 | 9.13 |
|  | Nair et al., 2018 | 4.12 | 1.79 | 9.49 |
|  | Labi et al., 2019 | 1.79 | 1.35 | 2.98 |
|  | Bunduki et al., 2019 | 5.11 | 1.46 | 17.83 |
|  | Birhanu et al., 2022 | 11.53 | 3.73 | 35.61 |
|  |  |  |  |  |
| Presence of peripheral and/or central vascular catheter | Salem et al., 2011 | 10.2 | 4.25 | 24.5 |
|  | Gadallah et al., 2014 | 1.9 | 1.42 | 2.57 |
|  | Labi et al., 2019 | 1.73 | 1.22 | 2.57 |
|  | Ayed et al., 2019 | 4.7 | 1.56 | 14 |
|  | Hassan et al., 2020 | 34 | 19.7 | 58.4 |
|  | Ketata et al., 2021 | 6.7 | 1.1 | 40 |
|  | Dawit et al., 2021 | 1.1 | 0.3 | 3.7 |
|  |  |  |  |  |
| Presence or urinary catheter | Melaku et al., 2012 | 18.9 | 2.54 | 15.7 |
|  | Iwuafor et al., 2016 | 5.38 | 0.11 | 29.86 |
|  | Labi et al., 2019 | 1.76 | 1.13 | 3.3 |
|  | Hassan et al., 2020 | 28.9 | 19.5 | 43 |
|  | Oumer et al., 2021 | 3.8 | 1.1 | 13.61 |
|  | Bunduki et al., 2021 | 8.3 | 2.24 | 30.7 |
|  | Flouchi et al., 2022 | 0.52 | 0.37 | 0.72 |
|  |  |  |  |  |
| Blood transfusion | Mukagendaneza et al., 2019 | 6.8 | 2.9 | 16.3 |
|  | Dramoski et al., 2016 | 8.1 | 3.9 | 16.6 |
|  | Alemye et al., 2021 | 4.2 | 2.35 | 6.08 |
|  | Birhanu et al., 2022 | 0.16 | 0.04 | 0.73 |
|  | Belay et al., 2022 | 2.78 | 1.13 | 6.86 |
|  | Mamo et al., 2017 | 6.75 | 2.47 | 18.49 |
|  |  |  |  |  |
| Hypertension and hypertensive disorders | Mpogoro et al., 2014 | 2.9 | 1.4 | 6.4 |
|  | Bizuayew et al., 2021 | 3.14 | 1.29 | 7.59 |
|  | Ayala et al., 2021 | 5.63 | 1.88 | 16.79 |
|  | Gomaa et al., 2021 | 1.19 | 0.92 | 2.11 |
|  | Molla et al., 2019 | 4.75 | 1.62 | 13.92 |
|  |  |  |  |  |
| Endotracheal intubation and mechanical ventilation | Gadallah et al., 2014 | 1.99 | 1.44 | 2.74 |
|  | Galal et al., 2016 | 1.79 | 1.08 | 2.99 |
|  | Ayed et al., 2019 | 17 | 3.2 | 90 |
|  | Abubakar et al., 2020 | 3.97 | 1.698 | 9.261 |
|  | Dawit et al., 2021 | 5.7 | 2.6 | 12.7 |
|  | Mohammed et al., 2022 | 3.46 | 1.44 | 9.81 |
|  | Belay et al., 2022 | 13.23 | 19.27 | 31.09 |
|  | Iwuafor et al., 2016 | 5.78 | 0.58 | 13.17 |
|  |  |  |  |  |
| Multiple vaginal examinations | Mpogoro et al., 2014 | 2.6 | 1.3 | 5.3 |
|  | Wodajo et al., 2017 | 8.59 | 1.74 | 42.23 |
|  | Ketema et al., 2020 | 1.88 | 1.71 | 3.2 |
|  | Lijaemiro et al., 2020 | 13.08 | 1.018 | 168.002 |
|  |  |  |  |  |
| Duration of labour >24 hours | Wodajo et al., 2017 | 6.78 | 2.54 | 18 |
|  | Gelaw et al., 2017 | 3.48 | 1.25 | 9.68 |
|  | Mamo et al., 2017 | 2.22 | 1.04 | 4.67 |
|  | Wendmagegn et al., 2018 | 6.06 | 1.676 | 21.949 |
|  | Bizuayew et al., 2021 | 3.48 | 1.49 | 8.09 |
|  | Ayala et al., 2021 | 4.12 | 1.01 | 32.19 |
|  | Gomaa et al., 2021 | 1.45 | 1.06 | 2.01 |
|  |  |  |  |  |
| Membrane rupture >12 hours | Wodajo et al., 2017 | 5.83 | 2.14 | 15.89 |
|  | Gelaw et al., 2017 | 3.68 | 1.13 | 11.96 |
|  | Mamo et al., 2017 | 5.99 | 2.75 | 13.02 |
|  | Wendmagegn et al., 2018 | 8.82 | 2.171 | 35.816 |
|  | Azeze et al., 2019 | 13.9 | 2.99 | 64.8 |
|  | Ketema et al., 2020 | 1.5 | 1.31 | 1.64 |
|  | Bizuayew et al., 2021 | 4.61 | 2.34 | 9.09 |
|  | Gomaa et al., 2021 | 3.99 | 3.11 | 4.74 |
|  | Di Gennaro et al., 2020 | 1.49 | 1.18 | 1.88 |
|  | Alemye et al., 2021 | 2.27 | 1.02 | 3.52 |
|  |  |  |  |  |
| Midline incision | Gelaw et al., 2017 | 5.73 | 2.05 | 16 |
|  | Mamo et al., 2017 | 4.95 | 1.71 | 13.32 |
|  | Molla et al., 2019 | 5.19 | 1.87 | 14.37 |
|  | Azeze et al., 2019 | 4.77 | 1.74 | 13.06 |
|  | Ketema et al., 2020 | 2.6 | 1.05 | 6.44 |
